# Complex emotion recognition system using basic emotions via facial expression, electroencephalogram, and electrocardiogram signals: a review

**DOI:** 10.3389/fpsyg.2026.1682883

**Published:** 2026-03-02

**Authors:** Javad Hassannataj Joloudari, Mohammad Maftoun, Bahareh Nakisa, Roohallah Alizadehsani, Meisam Yadollahzadeh-Tabari, Silvia Gaftandzhieva

**Affiliations:** 1 Department of Computer Engineering, Faculty of Engineering, University of Birjand, Birjand, Iran; 2 Department of Computer Engineering, Bab.C, Islamic Azad University, Babol, Iran; 3 Department of Computer Engineering, Technical and Vocational University (TVU), Tehran, Iran; 4 Department of Artificial Intelligence, Technical and Engineering Faculty, South Tehran Branch, Islamic Azad University, Tehran, Iran; 5 School of Information Technology, Faculty of Science Engineering and Built Environment, Deakin University, Geelong, VIC, Australia; 6 Institute for Intelligent Systems Research and Innovation, Deakin University, Waurn Ponds, VIC, Australia; 7 Faculty of Mathematics and Informatics, University of Plovdiv “Paisii Hilendarski”, Plovdiv, Bulgaria

**Keywords:** basic emotion, complex emotion, facial emotion recognition, meta-learning, physiological signals

## Abstract

The Complex Emotion Recognition System (CERS) deciphers complex emotional states by examining combinations of basic emotions expressed, their interconnections, and their dynamic variations. Through the utilization of advanced algorithms, the system provides profound insights into emotional dynamics, facilitating a nuanced understanding and customized responses. Achieving this level of emotional recognition in machines necessitates knowledge distillation and the comprehension of novel concepts akin to human cognition. The development of artificial intelligence systems for discerning complex emotions poses substantial challenges with significant implications for affective computing. Also, obtaining a sizable dataset for such systems is daunting due to the intricacies involved in capturing subtle emotions, necessitating specialized methods for data collection and processing. Incorporating physiological signals, such as electrocardiograms (ECG) and electroencephalograms (EEG), notably enhances CERS by furnishing insights into users' emotional states, improving dataset quality, and fortifying system dependability. This study presents a comprehensive review assessing the efficacy of machine learning, deep learning, and meta-learning approaches in both basic and complex emotion recognition using facial expressions, EEG, and ECG signals. Selected research papers offer perspectives on potential applications, clinical implications, and results of such systems, intending to promote their acceptance and integration into clinical decision-making processes. Additionally, this study highlights research gaps and challenges in understanding emotion recognition systems, encouraging further investigation by relevant studies and organizations. Lastly, the significance of meta-learning approaches in improving system performance and guiding future research is underscored, with potential applications in universities for advancing educational research, monitoring student well-being, and developing intelligent tutoring systems.

## 1 Introduction

Affective computing is an interdisciplinary domain integrating psychology, computer science, and cognitive science. A core component of this field is the automatic recognition of emotions, which enables machines to interpret human affective states and respond appropriately (Fan et al., 2023). Emotion recognition has attracted significant interest in both academia and industry due to its broad applications in human–computer interaction, gaming, and health monitoring for conditions such as Parkinson's disease, Alzheimer's disease, depression, and fall detection (Gandhi et al., 2023). Basic emotions such as happiness, sadness, fear, anger, surprise, and disgust are considered universal, innate, and associated with distinctive facial and physiological responses (Ekman, 1992; Ekman and Cordaro, 2011). In contrast, complex emotions, including jealousy, pride, and guilt, arise from combinations of basic emotions and higher-level cognitive processes, and are shaped by personal experiences and social context (Scherer, 2005; Izard, 2007).

Beyond the commonly used modalities such as facial expressions, EEG, and ECG, other physiological and behavioral channels also provide valuable cues for emotion recognition. These include electrodermal activity (EDA), which reflects sympathetic nervous system activity, and human gestures or subtle body movement patterns (Shukla et al., 2019; Zhu et al., 2019; Chen et al., 2023; Xia et al., 2025). Emotions manifest through multiple observable modalities, including physical expressions and physiological signals. Physical cues comprise facial expressions, vocal intonation, gait, and body posture, while physiological signals include EEG and ECG readings, galvanic skin response (GSR), and other biofeedback indicators.

Moreover, textual communication can also convey emotional content (Fan et al., 2023; Nokelainen et al., 2023; Oguz et al., 2023). Among these modalities, physiological signals have gained increasing importance due to their ability to capture internal affective changes influenced by the autonomic and central nervous systems. With the rise of the metaverse and the proliferation of wearable biosensors integrated into smart devices, emotion recognition through physiological data has become particularly relevant. EEG offers high temporal resolution for tracking brain activity in response to emotional stimuli (Nakisa et al., 2018; Nakisa, 2019), whereas ECG provides a reliable measurement of heart rate variability associated with emotional arousal (Rastgoo et al., 2019, 2024). Together, these signals complement facial expression analysis and improve the reliability of emotion recognition systems (Coskun, 2019; Kamble and Sengupta, 2023).

Despite these technological advancements, the ability to identify complex emotions remains significantly underdeveloped. The growing deployment of emotion-aware systems in mental health monitoring, social robotics, and metaverse applications demands models capable of interpreting nuanced affective states rather than only basic emotions. Without accurate recognition of emotions such as frustration, guilt, pride, or confusion, next-generation AI systems risk producing shallow or misleading interpretations of human behavior, making further research in this area both urgent and necessary.

Importantly, emotion-recognition deficits are not merely abstract computational challenges but well-documented and measurable clinical phenomena. Evidence from neurological and neurodegenerative disorders indicates that patients often exhibit systematic impairments in recognizing specific emotion categories. For example, individuals with Parkinson's disease show selective deficits in recognizing negative emotions such as fear and disgust, which have been linked to dysfunctions in fronto-striatal and limbic circuits (Hargrave et al., 2002; Chuang et al., 2022; Chiang et al., 2024). Similarly, studies on Alzheimer's disease and mild cognitive impairment report altered emotional processing and reduced sensitivity to facial and physiological affective cues (Güntekin et al., 2019). These findings highlight that emotion recognition performance can vary significantly across emotion classes and populations, underscoring the need for robust, multimodal, and adaptable AI-based emotion recognition systems that can generalize beyond healthy subjects and controlled laboratory settings.

Traditional emotion recognition systems often rely on a limited set of physiological measurements, which restricts their ability to capture the nuanced characteristics of complex emotions. Expanding the range of signals, particularly EEG and ECG, enhances understanding of the interplay between physiological systems and emotional responses (Majhi et al., 2024). However, due to the non-linear and non-stationary nature of physiological signals, careful feature extraction is required to achieve high accuracy (Booth et al., 2019; Chen et al., 2022).

Facial expression is another widely used modality for emotion recognition, representing the most direct and universal means of emotional communication (Li and Deng, 2020). Research indicates that facial expressions convey as much as 55% of emotional information, compared to 7% from verbal content and 40% from paralinguistic cues (Soleymani et al., 2015; Huang et al., 2019). Traditional machine learning algorithms, including SVM, MLP, Random Forest, and KNN, have been used extensively for facial emotion recognition (Raut, 2018), and have also been applied to physiological signals. Integrating multiple modalities, such as facial expressions with heart rate, skin conductance, and brain activity, can substantially improve recognition performance (Picard, 2000; Koelstra et al., 2011; Wang et al., 2014).

Deep learning approaches, particularly convolutional neural networks (CNNs), have demonstrated superior performance in many emotion recognition tasks (Schmidhuber, 2015; Giannopoulos et al., 2017). Despite their success, deep learning models require large labeled datasets and considerable computational resources, limiting their applicability in domains where data are scarce or expensive to obtain (Pouyanfar et al., 2018; Pramod et al., 2021; Ahmed et al., 2023). This issue is particularly relevant for complex emotions, which are difficult to label and less frequently represented in existing datasets (Grandjean et al., 2008; Calvo and D'mello, 2010; Khare et al., 2024). As a result, deep learning systems often struggle to generalize to real-world emotional scenarios that involve a blend of basic and complex affective states.

However, despite extensive efforts in emotion recognition research, a fundamental question remains unanswered: How can artificial intelligence effectively recognize complex emotional states using multimodal signals while overcoming data scarcity, high variability, and labeling ambiguity? This research specifically addresses this question by examining the role of meta-learning techniques in improving adaptability and performance in complex emotion recognition systems.

Despite significant progress in basic emotion recognition, existing research has largely overlooked complex emotions, which require integrating high-level cognition and multimodal physiological dependencies. Current studies either focus on limited emotion categories or rely on datasets that inadequately capture complex affective states.

To address these limitations, meta-learning strategies have been developed to enhance adaptability and performance in emotion recognition systems. Meta-learning enables models to learn from fewer samples, adapt quickly to new tasks, and handle noisy labels, making it well-suited for complex emotion recognition (Wang K. et al., 2020). Techniques such as few-shot learning (Ding et al., 2019), continual learning (He et al., 2019), and reinforcement learning (Li and Xu, 2020) have demonstrated promising results. Given that most widely used datasets such as MultiPie, SFEW, RaFD, JAFFE, and FER2013 mainly focus on basic emotions, meta-learning offers a pathway to overcome data scarcity and improve recognition of complex emotional states (Mellouk and Handouzi, 2020; Wu et al., 2023).

Despite recent advances, existing literature still focuses mainly on basic emotions and single-modality frameworks, creating a gap in understanding complex emotional states that emerge from cognitive processes and multimodal physiological interactions. To address this gap, this study proposes a structured research framework that links multimodal physiological signals, meta-learning adaptation, and complex emotion categorization, offering a more comprehensive perspective than prior surveys.

The main contributions of this paper are summarized as follows:

(1) Enhancing complex emotion recognition by multimodal fusion of facial expressions, EEG, and ECG signals through a meta-learning framework grounded in meta-learning principles;
(2) Presenting a focused survey on complex emotion recognition in AI that synthesizes multimodal and meta-learning–based approaches, and highlights key differences between basic and complex emotions, addressing gaps in existing review studies;
(3) Providing a unified taxonomy of complex emotion datasets along with a detailed discussion of dataset-related challenges, limitations, and open research directions;
(4) Reviewing and classifying meta-learning paradigms and their role in advancing complex emotion understanding and emotion classification.

### 1.1. Publication analysis and search results

**Figure 1:**
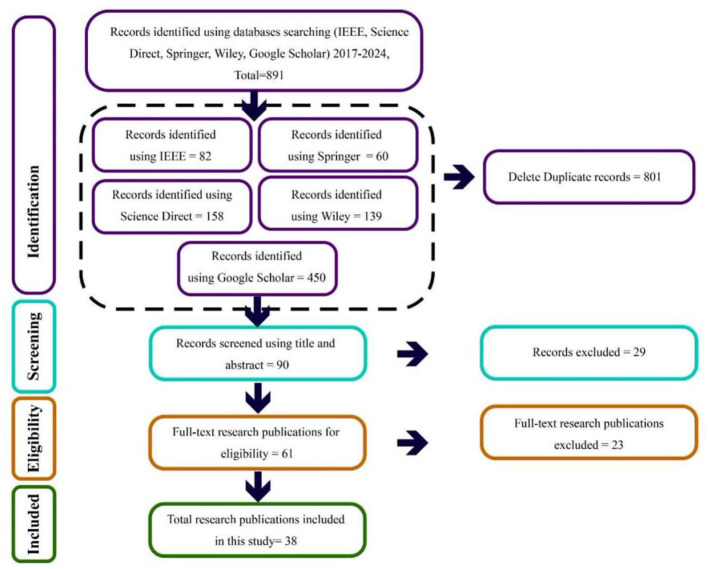
PRISMA flow diagram illustrating the identification, screening, eligibility assessment, and inclusion of studies in this review.

The review was conducted following PRISMA guidelines for systematic reviews, and a systematic search of the literature was conducted using five electronic databases, including IEEE Xplore, ScienceDirect, Springer, Wiley, and Google Scholar from 2017 to 2024. The search strategy yielded a total of 891 records, comprising 82 from IEEE, 60 from Springer, 158 from ScienceDirect, 139 from Wiley, and 450 from Google Scholar. A total of 90 studies remained for title and abstract screening, which resulted in 29 records excluded due to being off-topic, 61 full-text articles assessed for eligibility, 23 articles excluded due to not meeting the inclusion criteria, and 38 studies selected for qualitative synthesis in this review, after removing 801 duplicate and irrelevant records. The detailed study selection process is shown in Figure 1, following the PRISMA flow diagram. Also, a detailed database-specific search strategy, including full search strings and eligibility criteria, has been provided in the Supplementary materials.

### 1.2 Data mapping of included studies

**Figure 2:**
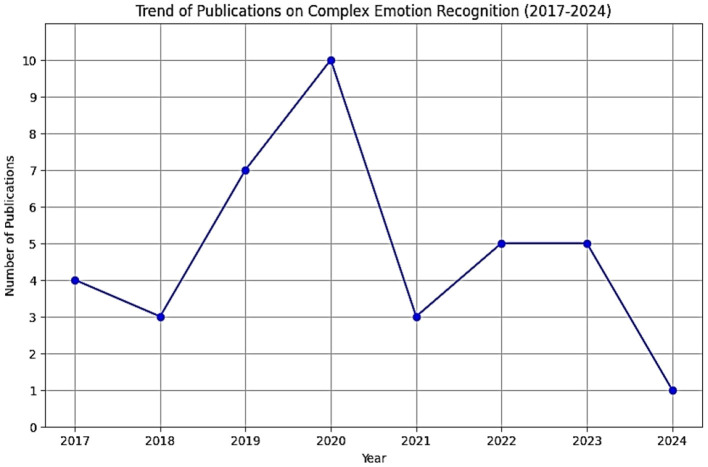
The trend of complex emotion recognition between 2017 and 2024.

In this section, we provide the research papers selected from various databases. The trend chart of publications on complex emotion recognition from 2017 to 2024 reveals a dynamic pattern, as shown in Figure 2.

From 2017 to 2020, there was a clear upward trend, with publications increasing from 4 to a peak of 10, indicating rising interest in the field. However, 2021 saw a sharp decline to 3 publications, possibly due to a temporary shift in research focus for resources. The numbers rebounded slightly in 2022 and 2023 with five publications each year, reflecting renewed efforts and interest. By 2024, the number dropped to its lowest at one publication, suggesting a potential winding down of major projects or shifting research priorities. Overall, the data highlights fluctuating engagement in complex emotion recognition research over the years.

**Figure 3:**
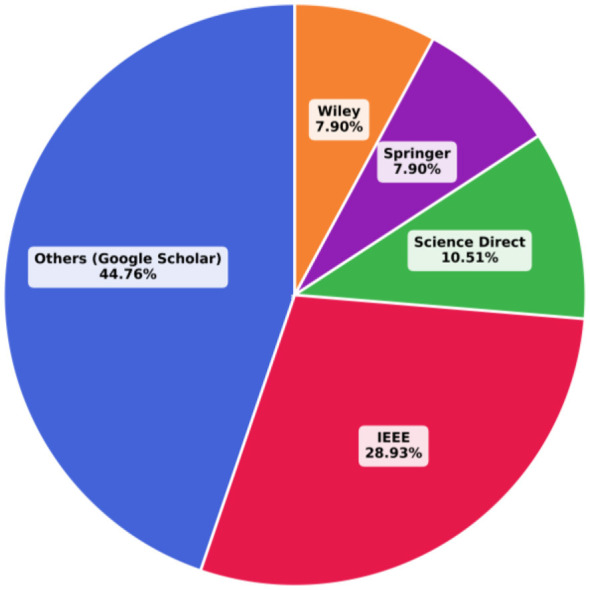
The allocated portion of each database in the complex emotion recognition topic.

According to Figure 3, the Google Scholar (others) database houses the most significant proportion of research papers in this field, accounting for nearly half (44.7%) of the total publications. The IEEE database follows, with 28.9% of the publications. Additionally, 10.5% of relevant studies were found on Science Direct. Lastly, while the Wiley and Springer databases each contributed only 7.89% of the research papers, they have nonetheless published valuable studies on complex emotion recognition systems.

To ensure comprehensive coverage and facilitate literature retrieval across multiple databases, the following keywords were employed during the search process: emotion recognition, complex emotion recognition, basic emotions, affective computing, physiological signals, EEG, ECG, EDA, facial expressions, human gestures, multimodal emotion recognition. These keywords guided the screening and selection of studies, allowing the review to capture both foundational works on basic emotions and recent advances in complex emotion recognition.

The next step of this study is structured as follows: Section 2 describes the types of emotions, their differences, and the emotion models. In section 3, the datasets used in scientific studies are reviewed and analyzed. Section 4 presents the preprocessing steps of developing a complex emotion recognition system. Section 5 focuses on the concepts and technologies of emotion recognition systems and also the limitations of traditional methods. In section 6, a review of related works is conducted. Section 7, the discussion and evaluation metrics are delivered. In Section 8, and last section are addressed into Open Research Challenges. Section 9 represents conclusions and future research directions.

## 2 Type of emotions, differences, and emotion models

### 2.1 Basic emotions

Emotions are brief emotional reactions that often occur automatically and have counterparts in other vertebrates. They are generally recognized worldwide and are commonly linked to specific physical reactions and facial expressions. Fear, anger, disgust, sadness, joy, and surprise are among the most frequently acknowledged basic emotions (Gu et al., 2019). Key features of basic emotions comprise:

1. Automaticity: Basic emotions are activated automatically in reaction to particular stimuli without conscious deliberation. For instance, the sight of a predator may trigger fear, while the presence of a loved one may trigger joy.
2. Distinctive Facial Expressions: Each basic emotion is tied to a distinct facial expression that is easily recognized across diverse cultures. For instance, a frown and squinting eyes are typically associated with anger, while a grin signifies happiness. Alongside facial signals, physiological metrics like EEG and ECG offer a more profound insight into emotional experiences. The EEG method captures the brain's activities tied to various emotional states, while the ECG technique evaluates heart rate variability and autonomic nervous system responses, both being notably affected by emotional excitement and stress. Collectively, these indicators present a more comprehensive view of emotion, merging external signals with internal physiological reactions.
3. Physiological Responses: Basic emotions come with specific physiological alterations in the body, such as variations in heart rate, breathing, and hormone levels. Fear, for example, initiates the “fight or flight” response, resulting in heightened heart rate and adrenaline release.
4. Evolutionary Roots: Basic emotions are thought to have distinct evolutionary functions tied to survival and procreation. Fear, for instance, aids individuals in reacting to dangers, while joy strengthens social connections and motivates actions that enhance overall well-being.

### 2.2 Complex emotions

Complex emotions are intricate and multifaceted emotional experiences that encompass a blend of basic emotions, cognitive processes, and social influences. Unlike basic emotions, which are generally widespread, complex emotions can vary significantly among individuals and societies (Black et al., 2020). Here are the primary characteristics:

1. Cognitive Components: Complex emotions entail substantial cognitive assessment and understanding of situations. Individuals may have to evaluate the context, their personal beliefs and values, and the perspectives of others to understand and experience intricate feelings fully. For instance, feelings of jealousy may emerge from interpreting a situation as a threat to a valued relationship.
2. Cultural and Individual Variation: In contrast to basic emotions, which are predominantly uniform, complex emotions can differ significantly among individuals and cultures. Cultural norms, values, and personal experiences have a substantial impact on shaping the experience and expression of complex emotions. For example, the way pride is felt and exhibited may vary across cultures.
3. Extended Duration: Complex emotions typically endure longer than basic emotions and may transform over time as new information and experiences are assimilated. For example, feelings of guilt may persist as individuals contemplate their actions and their repercussions.
4. Interpersonal Functions: Complex emotions often serve a vital role in managing social connections and navigating social standards. They assist individuals in comprehending and responding to others' emotions, negotiating social hierarchies, and upholding social ties. For instance, expressions of romantic love may entail a complex interplay of emotions like affection, desire, and commitment. Basic emotions and complex emotions differ in several key aspects. Firstly, regarding duration, basic emotions are typically short-lived and immediate, whereas complex emotions tend to persist for extended periods. Secondly, in terms of cognitive involvement, basic emotions often arise automatically with minimal cognitive processing, while complex emotions require significant cognitive appraisal and interpretation of situations. Thirdly, basic emotions have clear evolutionary functions primarily related to survival, whereas complex emotions have evolved to manage more sophisticated social interactions and relationships. Finally, basic emotions are universally recognized and expressed similarly across cultures, whereas complex emotions can vary significantly based on cultural and individual differences in expression and recognition (Zhu et al., 2019; Chaidi and Drigas, 2020; Weatherall and Robles, 2021).

### 2.3 Differences between basic and complex emotions

To further clarify the differences between basic and complex emotions, it is crucial to explore their cognitive and social implications more deeply. Basic emotions like joy, fear, anger, sadness, disgust, and surprise are often seen as universal and innate, arising automatically in response to stimuli with little conscious thought. These emotions are believed to have developed to fulfill specific survival purposes, such as fear activating fight-or-flight reactions to possible dangers, and disgust aiding individuals in avoiding harmful substances. Study by Ekman (1992) has shown that basic emotions are expressed and understood similarly across various cultures, hinting at a common evolutionary origin. In contrast, complex emotions such as guilt, shame, envy, pride, and love involve more intricate cognitive processes, often requiring self-reflection, comparison with others, and an understanding of societal norms and expectations. These emotions are not only enduring but also context-dependent, influenced by personal encounters, cultural upbringing, and the particular social setting. For example, feeling shame involves evaluating one's actions in relation to societal standards and anticipating others' judgments, which can differ significantly among cultures (Mesquita and Frijda, 1992; Keltner and Buswell, 1997). Research indicates that while the fundamental elements of these emotions might be universal, their expression, interpretation, and importance are molded by cultural context and individual distinctions (Mesquita, 2001).

Furthermore, complex emotions frequently result from combinations or interactions of basic emotions (Maiden and Nakisa, 2023) and are more likely to involve mixed sentiments or uncertainty. For instance, jealousy could blend fear (of losing a relationship) with anger (toward a perceived rival) and sadness (due to feeling unappreciated). This complex interplay of emotions showcases the sophisticated cognitive processes that underlie complex emotions, making them more challenging to investigate and comprehend compared to basic emotions (Parrott, 2001). Additionally, the influence of language in expressing and shaping complex emotions should not be underestimated; different societies possess specific terms and ideas that capture nuanced emotional states, emphasizing further the diversity in how these emotions are felt and conveyed (Wierzbicka, 1999). Basic emotions and complex emotions differ in several key aspects. Firstly, regarding duration, basic emotions are typically short-lived and immediate, whereas complex emotions tend to persist for extended periods. Secondly, in terms of cognitive involvement, basic emotions often arise automatically with minimal cognitive processing, while complex emotions require significant cognitive appraisal and interpretation of situations. Thirdly, basic emotions have clear evolutionary functions primarily related to survival, whereas complex emotions have evolved to manage more sophisticated social interactions and relationships. Finally, basic emotions are universally recognized and expressed similarly across cultures, whereas complex emotions can vary significantly based on cultural and individual differences in expression and recognition (Adolphs, 2017; Beall and Tracy, 2017; Rothman and Melwani, 2017; Milone et al., 2019; Šimić et al., 2021).

To provide a more precise and concise comparison, the key differences between basic and complex emotions are summarized in Table 1.

**Table 1:** The differences between basic and complex emotions.

**Aspect**	**Basic emotions**	**Complex emotions**
Duration	Short-lived, immediate	Longer-lasting, evolving over time
Cognitive involvement	Minimal, automatic response	Significant cognitive appraisal and interpretation
Evolutionary function	Survival and reproduction	Managing social interactions and relationships
Universality	Universally recognized across cultures	Varies with cultural and individual differences
Examples	Joy, fear, anger, sadness, disgust, surprise	Guilt, shame, envy, pride, love
Physiological correlates	Facial expressions, heart rate, EEG, ECG	Interaction of multiple basic emotions, language, context, social norms
Complexity	Simple, single emotion	Intricate, often a blend of emotions

After clarifying the distinctions between basic and complex emotions, it is also essential to consider the theoretical models used to represent emotional states in affective computing. Therefore, the following subsection introduces the two primary emotion modeling approaches used in the literature.

### 2.4 Emotion models

A clear definition of emotion is essential for establishing criteria in affective computing. Ekman initially formalized the understanding of emotions in the 1970s. Although numerous attempts have been made in psychology, neuroscience, philosophy, and computer science to classify emotions, no universal consensus exists. Nevertheless, two significant categories of emotion models are widely adopted: discrete (categorical) models and dimensional models (Wang Y. et al., 2022).

**Figure 4:**
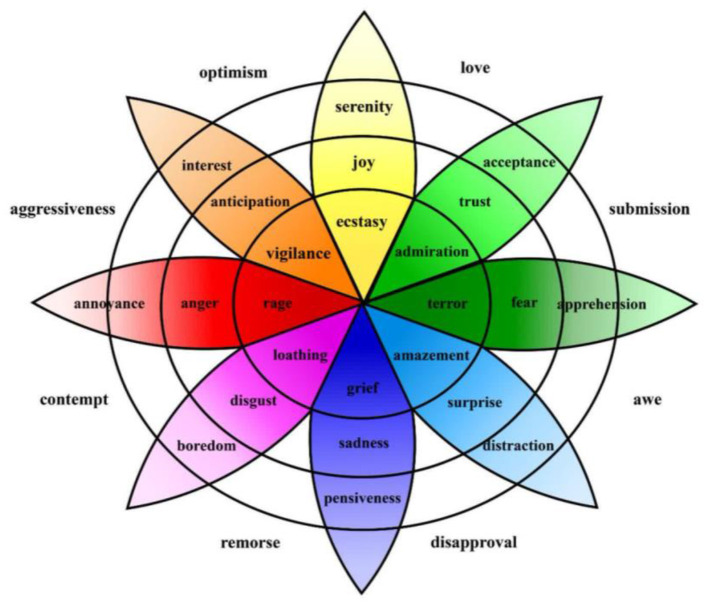
Wheel of emotions based on discrete models (Wang Y. et al., 2022).

As illustrated in Figure 4, discrete emotion models assume that individuals select one emotion from a predefined set of basic emotions. This representation, rooted in Ekman's early work, organizes emotions into distinct and easily identifiable categories.

**Figure 5:**
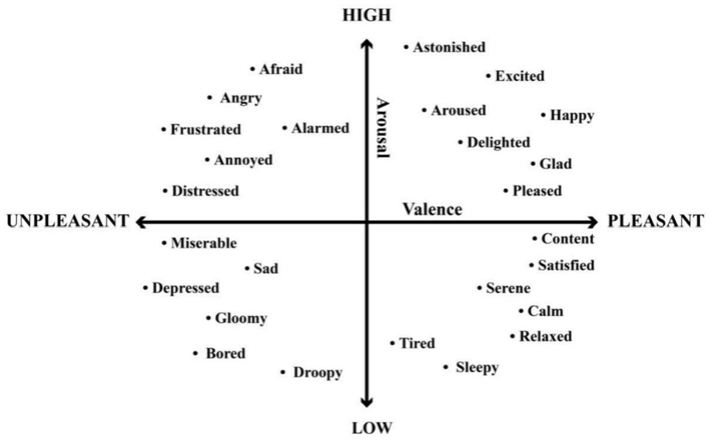
Emotions based on dimensional models (Sreeja and Mahalakshmi, 2017).

In contrast, dimensional models represent emotions continuously along quantitative dimensions, most commonly valence and arousal, as shown in Figure 5. Tools such as the Self-Assessment Manikin (SAM) and Feeltrace are typically used to assess these dimensions. SAM captures the static emotional state at a specific moment, whereas Feeltrace enables continuous monitoring of emotional variations over time (Sreeja and Mahalakshmi, 2017).

Both models highlight different aspects of emotional experience. The discrete model emphasizes clearly labeled emotional states, while the dimensional model captures the dynamic and nuanced nature of emotions. Together, they provide a comprehensive perspective essential for affective computing systems.

## 3 Datasets used in scientific studies

Datasets are fundamental for developing every AI-based system, encompassing the essential attributes necessary for facial emotion recognition, as well as for the analysis of EEG and ECG signals. These datasets facilitate the extraction and interpretation of results, and they are combined in advance to support the development of algorithms for recognizing facial emotions and analyzing physiological signals. They include a variety of data types such as static images, videos, or a combination of both (Chaubey and Pathrotkar, 2023; Vempati and Sharma, 2023). Additionally, some datasets include pictures taken in staged settings using laboratory-controlled environments or are provided by psychology centers. Moreover, datasets derived organically from real-world environments are also available.

### 3.1 Basic and complex emotion datasets

In this section, we categorize the most commonly used emotion recognition datasets into two main groups: basic emotion datasets, which focus on universally recognized emotions such as happiness, sadness, and anger, and complex emotion datasets, which aim to represent more subtle or compound emotional states. Additionally, some datasets encompass both basic and complex emotions, capturing a combination of these emotional expressions.

#### 3.1.1 Datasets with both basic and complex emotions

##### 3.1.1.1 CMED

Based on existing spontaneous micro-expression datasets such as CASME I (Zhao et al., 2025), CASME II (Yan et al., 2014), CAS(ME) (Qu et al., 2017), and Spontaneous Actions and Micro-Movements (SAMM) (Davison et al., 2016), researchers created the Compound Micro-Expression Database (CMED) (Zhao and Xu, 2019). These datasets mainly gather facial expression data rather than physiological signals. Due to their subtle features, it is challenging to analyze the motion track and characteristics of micro-expressions.

Consequently, generating compound micro-expression images presents numerous obstacles and limitations. The data in these datasets is captured using high-speed cameras that record slight facial movements at a high frame rate, typically ranging from 100 to 200 frames per second, enabling the detection of micro-expressions lasting between 1/25th and 1/5th of a second. Participants in these datasets engage in various tasks or are exposed to specific stimuli to evoke spontaneous emotional reactions, which are then documented (Yan et al., 2013, 2014; Davison et al., 2016; Zhao and Xu, 2019; Joloudari et al., 2024). The number of participants varies among the datasets.

For example, the CASME II dataset contains recordings from 26 individuals, while the SAMM dataset includes data from 32 subjects. The exact number of participants in the CMED may differ, but it usually encompasses a diverse group to ensure a wide array of facial expressions and emotions. These datasets cover a range of both basic and complex emotions. Common basic emotions captured include happiness, sadness, anger, fear, surprise, and disgust, which are universally understood and easily recognizable. Additionally, complex emotions like contempt, embarrassment, pride, and guilt are also recorded to offer a more thorough insight into human emotional expression.

##### 3.1.1.2 CASME

CASME is a micro-expression dataset developed by the Chinese Academy of Sciences (CAS). It was recorded at 60 frames per second and includes 180 videos from 19 subjects. Thirteen females and twenty-two males, with an average age of 22.03 years (σ = 1.60), participated in the study. Eight micro-expressions were recognized: amusement, sadness, disgust, surprise, contempt, fear, repression, and tension. The experiments focused on the top five classes (tense, disgust, happiness, surprise, and repression) with the most significant number of samples (Li et al., 2019). CASME builds upon previous datasets such as CAS-PEAL and SMIC, extending the available resources for studying subtle micro-expressions in a controlled environment.

##### 3.1.1.3 CFEE

This dataset contains 5,060 images of faces from 230 people, which labeled with 15 complex emotions and seven basic emotions (Guo et al., 2018). There are 21 different emotion categories in the CFEE dataset. Compound emotions, like delightfully surprised and angry surprised, comprise multiple basic component categories. The Facial Action Coding System was used to analyze of the obtained images. These 21 categories have distinct production processes that are nevertheless in line with the subordinate categories they represent (Kim and Kim, 2023).

#### 3.1.2 Basic emotions

##### 3.1.2.1 FER-2013

This dataset was introduced at the 2013 International Conference on Machine Learning (ICML) by Pierre-Luc Carrier and Aaron Courvill, the 2013 Facial Expression Recognition dataset (FER-2013) is available in the Kaggle dataset. The 35,887 grayscale 48 x 48-pixel images in the FER-2013 dataset remain in a spreadsheet with the pixel values of each image listed in a row of cells. After using Google to source images, they were categorized into various emotional classes, including surprise, anger, disgust, fear, happiness, neutral, and sadness. Upon the conclusion of the challenge, 3,589 images designated for private testing were added to the dataset, which initially contained 28,709 images for training and 3,589 images for public testing. Public test images are employed for a range of purposes in published research projects, each using the FER-2013 dataset distribution for individual training, validation, or test sets (Kusuma et al., 2020; Zahara et al., 2020).

##### 3.1.2.2 DEAP

The main emphasis of the DEAP dataset (Database for Emotion Analysis using Physiological Signals) lies in basic emotions. The DEAP dataset has been split into two sections (Tripathi et al., 2017). The first contains the ratings from 120 1-min music video excerpts that participants reviewed online using three criteria: arousal, valence, and dominance. The participants ranged in age from 14 to 16. The second accumulation of data comprises participant ratings, physiological recordings, and face films from an experiment in which 32 participants viewed a selection of the 40 music videos mentioned above. Every participant reviewed the videos as above, and physiological and EEG signals were recorded. Oval face videos were also captured for 22 subjects.

##### 3.1.2.3 BP4D+

The main focus of the BP4D+ dataset is on basic emotions. A large-scale, multimodal emotion dataset is known as BP4D+. The Facial Expression Recognition and Analysis (FERA) challenge of 2017 used the BP4D+ dataset. There are 140 participants total, aged 18–66, including 58 male and 82 female participants. Eight physiological signals are present in total: heart rate, respiration (rate and voltage), blood pressure (diastolic, systolic, mean, and raw), and electrodermal (EDA). Ten target emotions, including happiness, sadness, anger, disgust, embarrassment, astonished skepticism, fear, pain, and surprise are represented in the data for each subject (Fabiano and Canavan, 2019).

#### 3.1.3 Complex emotion datasets

##### 3.1.3.1 RAF-DB

29,672 real-world images of faces that were extracted from Flickr are included in RAF-DB. 315 talented annotators have labeled each of the RAF-DB's images, with roughly 40 independent annotators labeling each image. The single-label subset and the multi-label subset are the two distinct subsets found in RAF-DB (Greco et al., 2023).

##### 3.1.3.2 CEED

The Compound Emotion Expression Database (CEED) primarily focuses on capturing intricate emotions. Unlike datasets that center on fundamental emotions, CEED is tailored to document and examine emotional expressions that merge multiple basic emotions or encompass more subtle and socially influenced emotional states (Du et al., 2014). 480 images of eight young adult actors emulating nine complicated and six basic social-sexual emotional expressions are available in the CEED. There is some racial variety among the actors, who are both male and female. Almost 800 individuals independently scored images to confirm how the expression was perceived (Benda and Scherf, 2020). These datasets are crucial for advancing research in emotion recognition systems, encompassing a variety of basic and complex emotional states. It is noteworthy that in some cases, datasets primarily designed for recognizing basic emotions could also be utilized effectively for understanding and analyzing complex emotional states, thereby expanding the applicability and scope of these datasets (Maiden and Nakisa, 2023; Wang et al., 2023).

Although these datasets cover a broad spectrum of emotional expressions, a critical evaluation shows that the existing resources remain insufficient for building a reliable Complex Emotion Recognition System. Most available datasets are collected in controlled laboratory conditions, include a limited number of participants, and focus primarily on basic emotions. They rarely capture natural social interactions or real-world stimuli that typically evoke complex emotions. Moreover, truly multimodal datasets that simultaneously record EEG, ECG, EDA, facial dynamics, and behavioral cues are scarce, which limits the ability to model the rich cognitive mechanisms underlying complex affective states. Many datasets also lack demographic diversity and rely on single label annotation schemes, even though complex emotions are inherently overlapping and multi label. These limitations restrict the generalization ability of machine learning models and prevent meta-learning approaches from fully exploiting cross-task variability.

We proposed eight datasets corresponding to emotion recognition systems used in recent years, including facial expression, EEG, and ECG signals, which are presented in Table 2.

**Table 2:** The summarization of the mentioned datasets.

**References**	**Dataset name (complex/basic)**	**Emotions**	**Assessment types used**
Zhao and Xu (2019)	CMED—complex and basic emotion	Happiness, disgust, fear, anger, sadness, surprise, happily surprised, sadly surprised, fearfully surprised, angrily surprised, disgustedly surprised, happily disgusted, sadly fearful, sadly angry, sadly disgusted, fearfully angry, fearfully disgusted, angrily disgusted	Not specified
Takalkar and Xu (2017)	CASME—complex and basic emotion	Contempt, disgust, fear, happiness, regression, sadness, surprise, tense	Self-report ratings
Mavani et al. (2017)	CFEE—complex and basic emotion	Angry, fearful, disgusted, surprised, happy, sad and neutral	Facial action coding system
Han et al. (2023)	FER-2013—basic emotion	Happy, sad, angry, fear, surprise, disgust, and neutral	Not specified
Khateeb et al. (2021)	DEAP—basic	Signal-based (EEG)	Self-report ratings, physiological recordings, EEG
Guerdelli et al. (2022)	BP4D+–basic	Happiness or amusement, surprise, sadness, startle or surprise, skeptical, embarrassment, fear or nervous, physical pain, angry and disgust	Self-report ratings, physiological recordings, EEG
Yan et al. (2022)	RAF-DB—complex	Neutral, happy, surprise, sad, anger, disgust, fear	Not specified
Benda and Scherf (2020)	CEED—complex	Six basic expressions (angry, disgusted, fearful, happy, sad, and surprised) and nine complex expressions (affectionate, attracted, betrayed, brokenhearted, contemptuous, desirous, flirtatious, jealous, and lovesick)	Self-report ratings

Based on the limitations identified across current emotion databases, a featured direction for future dataset development is the creation of a large-scale ecological multimodal resource that captures naturally occurring complex emotions. Such a dataset should include synchronized EEG ECG EDA facial video body gestures speech and contextual information while participants engage in fundamental social interactions or immersive experiences. It should also include multi-label annotations reflecting blended emotional states rather than single category labels. A diverse participant pool, longer recording durations, and culturally varied emotional scenarios would further enhance its value for CERS. Developing this type of comprehensive multimodal dataset would significantly advance the generalization and robustness of meta-learning-based complex emotion recognition models.

## 4 Preprocessing in CEMRS

Preprocessing in complex emotion recognition systems (CEMRS) plays a pivotal role in ensuring the quality, consistency, and interpretability of multimodal data. As emphasized in recent multimodal affective computing studies, preprocessing acts as the foundation upon which all subsequent modeling stages depend. Variability across sensors, differing temporal resolutions, and susceptibility to noise can significantly distort the representation of complex emotions if not adequately handled. Therefore, the preprocessing stage directly determines how well subtle, slow-evolving, and context-dependent emotional states can be captured, preserved, and later learned by machine learning and deep learning models (Ahmed et al., 2023; Wu et al., 2025).

Unlike basic emotions, which are often short, distinct, and easily captured, complex emotions emerge through longer temporal patterns and involve subtle physiological and physical cues. Therefore, preprocessing in CEMRS must address noise removal, temporal alignment, cross-modality normalization, artifact correction, and feature-scale harmonization across modalities such as facial video, EEG, and ECG signals. To provide a comprehensive perspective aligned with recent literature, this section is organized into several subsections. First, the preprocessing challenges associated with physical cues (Section 4.1) and physiological cues (Section 4.2) are reviewed. Then, the critical aspects of cross-modality integration and synchronization (Section 4.3) are discussed, followed by a conceptual unified preprocessing pipeline derived from existing studies (Section 4.4). Finally, issues related to data imbalance and standardization (Section 4.5) and empirical evidence illustrating the impact of preprocessing on system performance (Section 4.6) are presented.

### 4.1 Physical cues preprocessing

This section focuses on the preprocessing difficulties related to physical cues, specifically facial expressions obtained via image processing. It delves into the intricacies of preparing facial images to derive significant features that represent various emotional states. Major challenges encompass managing fluctuations in lighting conditions, facial angles, and the range of facial expressions. Furthermore, it highlights the necessity for sophisticated techniques in feature extraction to effectively capture the subtleties of intricate emotions (Schulze et al., 2017). Initial stages involve detecting and aligning faces to ensure consistent positioning and orientation of facial features in images. Following face detection, normalization methods are utilized to counteract lighting variations by adjusting brightness, contrast, and color balance for standardization. Next, noise reduction techniques like Gaussian blurring or median filtering are applied to minimize unwanted image artifacts that could disrupt emotional cue recognition.

Feature extraction is then carried out to capture important facial attributes crucial for emotion recognition, including identifying facial landmarks and extracting texture descriptors from facial regions. Given that feature vectors often consist of intricate data, dimensionality reduction techniques like Principal Component Analysis (PCA) or t-Distributed Stochastic Neighbor Embedding (t-SNE) are applied to decrease computational complexity and prevent overfitting. Through these preprocessing steps, facial expression recognition systems can enhance their accuracy, resilience, and ability to generalize in emotion classification tasks. This thorough preprocessing process ensures that the recognition system is well-prepared to provide consistent and precise results across various input images, enabling applications in affective computing, human-computer interaction, and physical research (Samadiani et al., 2019; Li et al., 2020).

While these steps are effective for basic emotion recognition, complex emotions require more specialized preprocessing strategies. Basic emotions typically manifest through distinctive and rapid facial muscle activations, enabling frame-level analysis. In contrast, complex emotions such as guilt, embarrassment, pride, or admiration develop more slowly and involve combinations of micro-expressions and context-dependent behavior. Therefore, preprocessing for complex-emotion facial analysis must incorporate temporal smoothing, stable landmark tracking, sequence-level normalization, and dynamic alignment to preserve emotional transitions over time. This distinction highlights that a single uniform preprocessing approach is insufficient for CEMRS and must instead be tailored to the temporal and contextual nature of complex emotions.

Furthermore, because complex emotions evolve over extended time intervals, preprocessing workflows increasingly incorporate temporal embedding or sequence-preserving strategies. These include techniques such as sliding-window smoothing, long-term landmark stability estimation, and frame-to-sequence normalization, which help retain the dynamic transitions that characterize emotions such as pride, guilt, or admiration. Such temporal-aware preprocessing has been shown to enhance downstream sequence models, including Long Short-Term Memory (LSTM) and temporal CNNs, by providing more coherent and contextually aligned facial representations (Do et al., 2021; Sanchez et al., 2021).

### 4.2 Physiological cues preprocessing

This section addresses the difficulties associated with preprocessing physiological cues, such as those derived from EEG and ECG signals, through signal processing. It encompasses the preprocessing procedures needed to cleanse and filter physiological signals, extract relevant features, and alleviate artifacts to capture the underlying emotional states precisely. Challenges in this field may involve noise reduction, artifact elimination, feature extraction from intricate physiological signals, and ensuring the dependability and precision of the processed data for emotion recognition (Yang et al., 2021).

The preprocessing workflow involves several essential steps tailored to optimize EEG and ECG signals for effective analysis. Firstly, artifact removal is imperative to eliminate noise and unwanted interference from the signals. EEG signals, for instance, are susceptible to various artifacts like eye blinks, muscle movements, and environmental electrical noise, while ECG signals can be affected by muscle activity and movement artifacts. Techniques such as independent component analysis (ICA) and adaptive filtering are commonly used to mitigate these artifacts, ensuring that the extracted signals accurately represent the underlying neural and cardiac activity. Following artifact removal, signal segmentation is performed to divide continuous EEG and ECG recordings into temporally meaningful epochs, often aligned with emotional stimuli. After segmentation, feature extraction is conducted to capture relevant characteristics: EEG features may include spectral power, coherence, and hemispheric asymmetry, while ECG features may encompass heart rate variability (HRV), amplitude variations, and RR-interval distributions. Dimensionality reduction methods such as PCA and wavelet decomposition help reduce feature complexity while preserving meaningful physiological information.

≪When EEG and ECG signals are used jointly within a multimodal framework, cross-signal normalization becomes a crucial preprocessing step. Differences in amplitude ranges, sampling densities, and temporal drift can introduce cross-modality bias, degrading fusion performance. One effective approach is stratified normalization, which aligns signal statistics across participants by normalizing per participant and feature, thereby reducing inter-subject variability while preserving emotion-related information. This method has been shown to improve cross-subject emotion classification accuracy significantly (Fdez et al., 2021).

By implementing these preprocessing steps, EEG- and ECG-based emotion recognition systems improve accuracy, robustness, and generalization capabilities (Shoka et al., 2019; Dora and Biswal, 2020; Rashmi and Shantala, 2022).

### 4.3 Cross-modality integration and synchronization

A main challenge in CEMRS is integrating multimodal sources with inherently different sampling rates and temporal characteristics. EEG typically ranges between 128 and 1,024 Hz, ECG between 250 and 500 Hz, while facial video operates at 25–30 fps. Without proper synchronization, multimodal fusion may introduce temporal drift, weakening emotional interpretation. Methods such as cross-correlation, dynamic time warping (DTW), and event-based alignment are effective in matching emotional episodes across modalities. Empirical studies show that accurate synchronization improves complex emotion recognition performance by 10–20%, emphasizing its crucial role in the preprocessing pipeline.

These findings are echoed in recent multimodal emotion recognition research that explicitly tackles temporal misalignment and cross-modal synchronization. For instance, Li and Chen proposed a cross-modal alignment and fusion model for EEG–visual emotion recognition, based on a hybrid attention mechanism that aligns EEG features and video frames to correct for synchronization issues (Li and Chen, 2025).

Moreover, emerging methods such as PhysioSync use temporal and cross-modal contrastive learning, inspired by physiological synchronization, to improve alignment between EEG and peripheral signals (Cui et al., 2025). These approaches demonstrate that modeling fine-grained temporal consistency can significantly boost performance in multimodal emotion recognition.

### 4.4 Unified preprocessing pipeline

**Figure 6:**
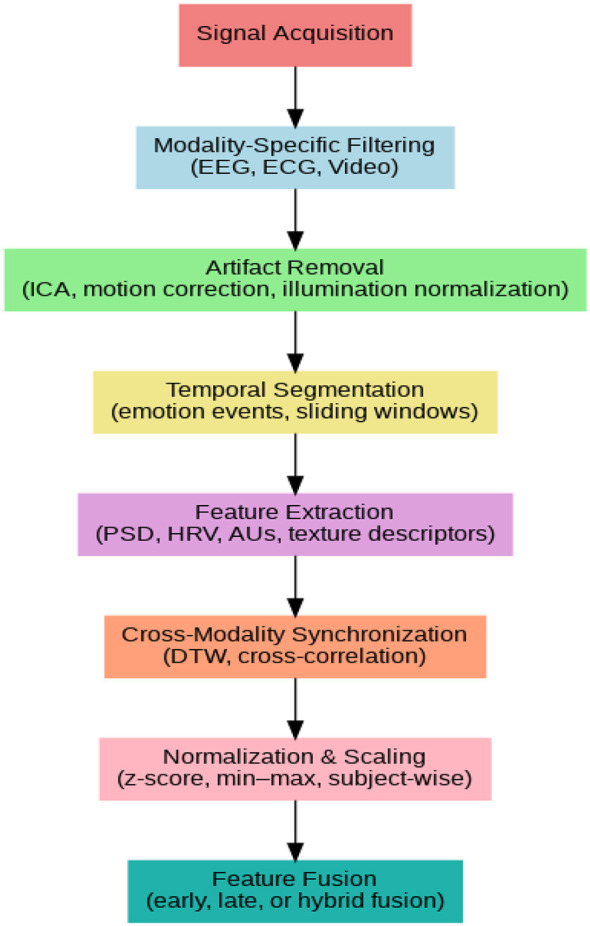
General preprocessing pipeline for multimodal CEMRS.

A unified preprocessing pipeline is essential for summarizing the common steps reported across multimodal CEMRS studies and for clarifying how different preprocessing components interact within a complete recognition workflow. As shown in Figure 6, existing research generally follows a high-level sequence that includes signal acquisition, modality-specific filtering, artifact removal, temporal segmentation, feature extraction, cross-modality synchronization, normalization, and multimodal feature fusion. While the exact implementation of these stages varies across studies, the overall structure remains consistent and reflects the shared challenges associated with preparing heterogeneous data sources. To provide a complementary perspective, Table 3 offers a comparative overview of preprocessing methods that have been systematically reviewed or empirically evaluated for EEG, ECG, and multimodal emotion recognition. Each row references a peer-reviewed study and highlights both the advantages and limitations of the approach. When available, improvements in classification performance are reported quantitatively; in other cases, the table reflects consistent qualitative trends observed in the literature. By explicitly linking each claim to a verified source, the table avoids unsupported or illustrative performance ranges.

**Table 3:** Comparative summary of preprocessing techniques across modalities.

**Reference**	**Modality**	**Preprocessing/feature extraction**	**Advantages**	**Limitations**	**Reported performance/evidence**
Erat et al. (2024)	EEG	Band-pass filtering	Reduces low- and high-frequency noise; widely used to enhance EEG signal quality before feature extraction	Effects on affect-related EEG components are not explicitly quantified; filter design and cutoff frequencies vary across studies	Common preprocessing step; improvement in signal quality and classifier stability is heterogeneous across studies
Grilo Jr et al. (2023)	EEG	ICA/BSS-based artifact removal using MI and Epanechnikov kernel	Removes ocular and muscle artifacts; improves EEG signal quality for emotion classification	Method depends on algorithm complexity and component identification	Improves classification performance; effects vary across datasets
Sepúlveda et al. (2021)	ECG	Wavelet-based feature extraction and denoising	Extracts multi-scale time–frequency features; enhances separability of emotional patterns; improves classification accuracy	Increases feature complexity and computational cost	Classification accuracy: 88.8% (valence), 90.2% (arousal), 95.3% (2-D)
Wang and Wang (2025)	Multimodal (EEG + ECG)	Denoising (EEG and ECG), feature extraction, multimodal fusion	Combines EEG and ECG signals; deep learning fusion enhances discriminative power	Effect varies by dataset and feature space; requires careful preprocessing	Reported high classification accuracies, e.g., 95.95% on valence dimension; significant improvement over unimodal approaches
Kang and Lee (2025)	Multimodal (EEG + ECG)	EEG functional connectivity + ECG temporal feature extraction + parallel fusion	Combines EEG and ECG features; improves discriminative power for emotion classification	Computationally complex; requires careful model training	In subject-dependent setting, proposed method outperformed baseline by +10.41% for valence and +9.54% for arousal

This pipeline ensures temporal coherence, cross-modality compatibility, and standardized feature preparation before model training.

As detailed in Table 3, each modality employs specific preprocessing strategies designed according to its signal characteristics and susceptibility to noise. EEG preprocessing often involves band-specific filtering and artifact removal techniques, such as ICA, which generally enhance signal quality and feature stability, though the degree of improvement varies across studies and datasets. ECG preprocessing primarily targets denoising while preserving HRV-related information, with performance gains typically described qualitatively rather than as precise numeric measures. In facial video preprocessing, geometric alignment and illumination normalization are applied to maintain consistent visual representations; however, effectiveness may vary depending on occlusion, lighting, and dataset conditions. For multimodal systems, normalization methods and temporal alignment techniques, including Z-score scaling and DTW, facilitate the integration of heterogeneous data, with their impact differing across contexts. Collectively, the trends summarized in Table 3 indicate that preprocessing has a significant influence on model outcomes, but the magnitude of improvement is heterogeneous, reflecting variations in datasets, modalities, and evaluation protocols rather than fixed numeric ranges.

### 4.5 Data imbalance and standardization

A widely reported challenge in multimodal complex emotion datasets is the severe imbalance among emotion categories. Complex emotions such as shame, guilt, pride, or admiration often appear far less frequently compared to basic emotions, causing models to become biased toward majority classes and leading to reduced F1-scores for minority categories. Several studies across the literature have addressed this issue through imbalance-aware preprocessing strategies, including oversampling approaches such as SMOTE and ADASYN, class-balanced weighting, subject-wise normalization, and robust scaling techniques. According to empirical findings, these preprocessing adjustments can yield substantial performance improvements—sometimes up to 18% for minority emotion classes—highlighting the importance of imbalance handling in CEMRS studies.

### 4.6 Empirical evidence of preprocessing effectiveness

Across the literature, preprocessing has consistently been identified as a key determinant of performance in CEMRS. Numerous studies demonstrate that artifact removal and signal cleaning significantly enhance system accuracy: ICA-based EEG preprocessing has been shown to improve recognition performance by 10–15%, ECG filtering contributes improvements of 5–8%, and illumination normalization in facial video preprocessing increases CNN classification accuracy by 7–10%. These findings, reported across diverse datasets and modalities, underline that preprocessing is not a peripheral step but a central component shaping the reliability of multimodal emotion recognition.

## 5 Challenges and solutions in emotion recognition models

Emotion recognition has witnessed significant advancements through machine learning and deep learning approaches. Most studies have primarily focused on recognizing basic emotions using predefined features extracted from facial expressions, physiological signals, or other modalities. However, human emotions are inherently complex, often involving subtle, context-dependent variations that challenge traditional models. While conventional machine learning and deep learning methods can achieve reasonable performance on basic emotions, they struggle to capture the richness and diversity of complex emotional states. Moreover, these models often face limitations such as overfitting on limited datasets, dependency on handcrafted features, and insufficient adaptability to individual differences.

To address these challenges, recent research has explored advanced solutions, particularly meta-learning, which allows models to “learn how to learn” and rapidly adapt to new emotional contexts even with limited data. Complementary approaches, including continual learning, few-shot learning, label noise handling, and reinforcement learning, further enhance model robustness, generalization, and adaptability. This section provides a comprehensive overview of these challenges and solutions, presenting a structured taxonomy of emotion recognition models and their respective methodologies.

### 5.1 Challenges

Despite significant advances in machine learning and deep learning, recognizing human emotions remains a challenging task. These models often struggle to capture the complexity, context-dependence, and subtle variations of emotional states. As a result, traditional approaches face challenges in accurately understanding and generalizing complex emotions across different individuals and situations.

#### 5.1.1 Inability to capture complex emotional states

Traditional machine learning models, including deep learning models, often rely on predefined features and lack the flexibility to capture the nuances and subtleties of complex emotional states. As a result, they may struggle to distinguish between similar emotional expressions or interpret the context-dependent nature of complex emotions (Hassouneh et al., 2020).

#### 5.1.2 Dependency on handcrafted features

Many traditional machine learning and deep learning approaches require handcrafted features to be extracted from the data. These features may not fully represent the multidimensional nature of complex emotions, leading to a limited ability to generalize across different emotional contexts (Tzirakis et al., 2017; Côté-Allard et al., 2020).

#### 5.1.3 Limited adaptability to individual differences

Traditional machine learning and deep learning models may not be adaptable enough to account for individual differences in expressing and experiencing emotions. They often rely on generic models that do not adequately capture the variability and subjectivity of emotional responses among different individuals (Zhao et al., 2019).

#### 5.1.4 Overfitting and generalization issues

Traditional machine learning and deep learning models may be prone to overfitting when trained on limited datasets, resulting in poor generalization to unseen data. This is particularly problematic in the case of complex emotions, where the variability and diversity of emotional expressions can be significant (Kanjo et al., 2019).

### 5.2 Solutions

#### 5.2.1 Meta-learning

**Figure 7:**
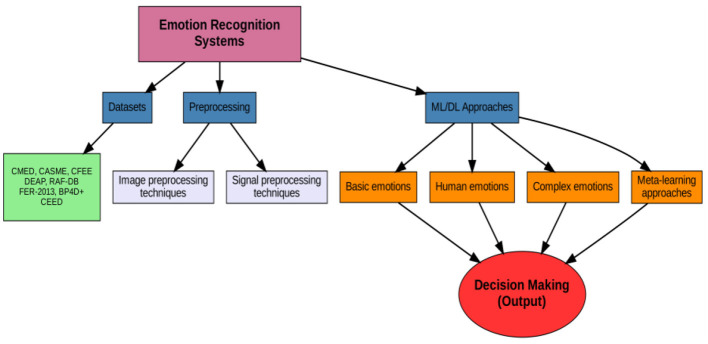
The taxonomy of emotion recognition systems.

In the face of these challenges, meta-learning offers promising approaches to enhance the capabilities of complex emotion recognition systems. Meta-learning, which focuses on learning how to learn, can provide a framework for adapting traditional machine learning and deep learning models to handle complex emotional states better. By leveraging meta-knowledge acquired from a diverse range of emotional contexts, meta-learning algorithms can enhance the adaptability and generalization capabilities of emotion recognition systems. This approach enables models to learn from previous experiences and rapidly adapt to new emotional contexts, thereby improving their performance in recognizing complex emotions (Gandhi, 2022; Zhou et al., 2021). Most studies have presented machine learning and deep learning techniques for recognizing emotion based on basic features, but emotions are always complex. Few studies have worked on complex emotions based on basic features for recognition. In this scenario, another approach called meta-learning could play a vital role in these types of tasks. Figure 7 illustrates our proposed taxonomy for emotion recognition systems.

According to Figure 7, methods are divided into basic and complex for introducing emotion recognizer systems based on machine learning, deep learning (1), and meta-learning (2).

1. Machine Learning and deep learning approaches for basic emotion recognition

Based on Figure 6, machine learning approaches such as SVM, Random forest, KNN, Naive Bayes, etc. were utilized as feature selection and prediction tasks for emotion recognition (Sepúlveda et al., 2021). Deep learning methods, such as recurrent neural networks (RNNs) and CNNs, have made significant strides in the field of computer vision in recent decades. These techniques based on deep learning have been used for problems related to recognition, classification, and feature extraction. By enabling “end-to-end” learning directly from input images, a CNN's primary benefit is to eliminate or greatly reduce reliance on physics-based models and/or other pre-processing approaches. Due to these factors, CNN has produced cutting-edge outcomes in several domains, such as emotion recognition tasks based on face expression, ECG, and EEG signals (Ko, 2018; Islam et al., 2021).

In addition to conventional CNN and RNN architectures, hybrid spatio-temporal deep models such as recurrent convolutional networks (RCN) have been proposed to leverage both spatial and temporal information in facial expression sequences (Xia et al., 2020). For example, Xia et al. (2019) introduced a deep recurrent convolutional network for micro-expression recognition, where recurrent convolutional layers extract spatial features from each frame while modeling their temporal evolution. Kim et al. also applied a convolutional-recurrent neural network (CRNN) for driver facial expression analysis, demonstrating the effectiveness of combining CNN and RNN layers for capturing facial dynamics (Kim et al., 2020). Moreover, Du et al. (2024) used attention-based 3D convolutional recurrent neural networks (CRNN) to capture subtle facial dynamics over time.

2. Meta-Learning for Complex Emotion Recognition

Meta-learning, commonly referred to as “learning to learn,” is the process through which AI models acquire the ability to swiftly adjust to new environments or tasks while working with limited data. This capability is especially beneficial in the realm of complex emotion recognition, where challenges such as data scarcity and variability across different contexts frequently arise. Meta-learning comprises various methodologies: optimization-based, model-based, and metric-learning-based techniques (Wang W. et al., 2022). Optimization-based techniques, including Model-Agnostic Meta-Learning (MAML), Reptile, and Almost No Inner Loop (ANIL), focus on identifying optimal initialization parameters that allow models to converge on new tasks with minimal modifications quickly. These techniques offer flexibility and adaptability across a variety of emotion recognition scenarios by effectively learning from a handful of examples (Nichol et al., 2018; Feng et al., 2022). Model-based techniques, such as recurrent and convolutional neural networks, integrate meta-learning principles directly into their architecture. These models are crafted to adjust to new tasks internally; however, they may encounter difficulties in generalizing to more complex or diverse emotional contexts due to their streamlined optimization processes. Metric-learning-based techniques, like ProtoNet, RelationNet, and MatchingNet, depend on learning embedding functions that transform data into a space where classification can be performed using similarity metrics. These non-parametric approaches prove effective for emotion recognition as they facilitate rapid learning from sparse data by exploiting the relationships among data points (Nguyen et al., 2023). In the subsequent sections, we will examine how meta-learning principles are implemented in Continual Learning, Few-shot Learning, Label Noise management, and Reinforcement Learning, all of which enhance the proficiency of models in recognizing intricate emotions: Continual Learning guarantees that models can adapt to new emotional tasks over time without losing knowledge of previously learned tasks, which is essential in dynamic environments where emotions are subject to change.

Few-shot Learning confronts the obstacle of gaining insights from a small quantity of emotion-tagged information by empowering models to extrapolate proficiently from a tiny set of samples. Label Noise management involves techniques for addressing noisy or inaccurate labeling of emotional data, thereby improving the model's robustness and reliability in real-world emotion recognition applications. Reinforcement Learning aids in optimizing emotion recognition policies through trial and error, allowing models to enhance their performance by engaging with their surroundings. These methodologies underscore the importance of meta-learning in the advancement of complex emotion recognition. Through boosting the adaptability, resilience, and productivity of emotion recognition technologies, meta-learning is essential for confronting the intrinsic challenges that arise in this section (Wang W. et al., 2022; Wang et al., 2024).

#### 5.2.2 Continual learning and few-shot learning

Continual learning involves compiling research and methods to tackle the challenge of learning in scenarios where knowledge integration across endless streams of data needs to be considered, especially when the data distribution fluctuates over time (Parisi et al., 2019; Lesort et al., 2020). In the realm of continual learning, models are crafted to acquire knowledge from new data while preserving previously gained knowledge, a task made difficult by catastrophic forgetting, a phenomenon where new learning can disrupt and overwrite previously absorbed information. To combat this issue, continual learning strategies often incorporate replay techniques, where a model is regularly trained on a combination of fresh data and selected samples of past data. These replay methods play a vital role in achieving the perfect equilibrium between stability, the maintenance of existing knowledge, and adaptability, effectively integrating new information.

**Figure 8:**
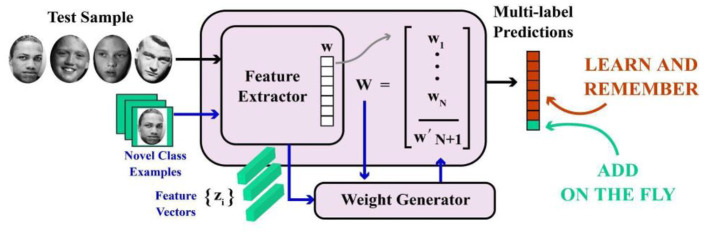
Continual learning in complex emotion recognition systems.

Furthermore, additional approaches like regularization techniques, which discourage alterations to crucial weights, and parameter isolation methods, which designate specific parts of the model for different tasks, are utilized to alleviate catastrophic forgetting and boost the performance of continual learning systems (Chang et al., 2021). The architecture of continual learning is indicated in Figure 8.

**Figure 9:**
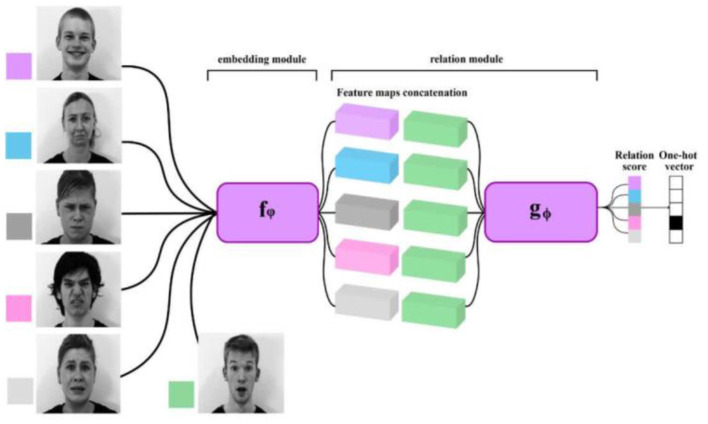
Few-shot learning in complex emotion recognition systems.

According to Figure 9, few-shot learning is the concept for developing an expanding algorithm from an insignificant sample set. Few-shot learning for facial emotion recognition has been established to decrease the intraclass distance and enhance the interclass distance (Tian et al., 2024).

#### 5.2.3 Handling label noise in complex emotion recognition

**Figure 10:**
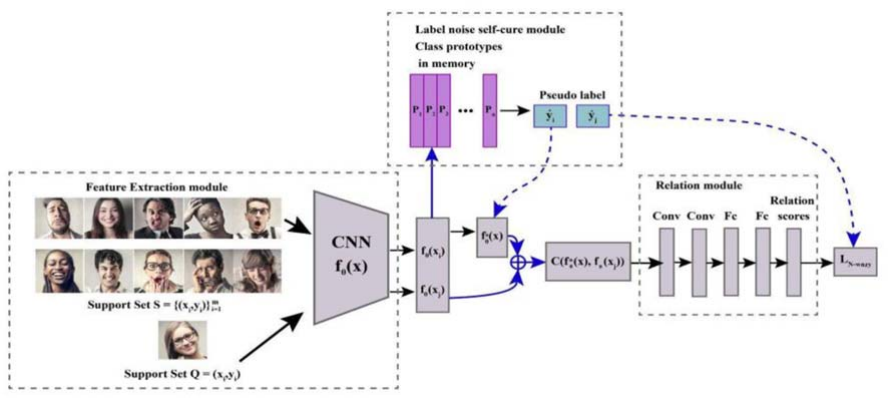
Architecture of meta-learning based on label noise.

Label noise is a prevalent issue in real-world datasets caused by several factors, including the expense of the labeling process and the challenge of accurately classifying data. In the field of affective computing for complex emotion recognition, the use of noisy labels addresses several challenges inherent in interpreting facial expressions. Concerning the subjective nature of human emotions, different annotators frequently provide diverse interpretations, leading to problems in labeling. Noisy labels help manage this ambiguity by allowing models to learn from a distribution of possible labels instead of a single, potentially erroneous one, thereby fostering more robust representations. Moreover, Figure 10 demonstrates that integrating label noise during training also enhances model robustness by leveraging techniques like label distribution learning, which improves generalization and mitigates the impact of incorrect labels. This approach is particularly beneficial for handling the complexity of real-world facial expressions, which often involve mixtures of basic emotions. Noisy labels mirror this variability and help models distinguish subtle emotional nuances. Additionally, methods like Face-Specific Label Distribution Learning (FSLDL) create augmented training samples with label distributions, broadening the range of expressions and viewpoints captured, thus enhancing the model's ability to generalize to new data. To prevent overfitting to noisy samples, techniques such as rank regularization and discriminative loss functions are employed, ensuring that the model focuses on more reliable samples and maintains overall performance. By addressing ambiguity and subjectivity, improving robustness, handling complex expressions, enhancing training with augmented data, and reducing overfitting, noisy labels significantly contribute to the advancement of complex emotion recognition systems in affective computing (Wu et al., 2018; Zeng et al., 2018; Karimi et al., 2020; Sarkar and Etemad, 2020; Wang Z. et al., 2020; Saganowski et al., 2022; Song et al., 2022; Yan et al., 2022).

#### 5.2.4 Reinforcement learning for complex emotion recognition

**Figure 11:**
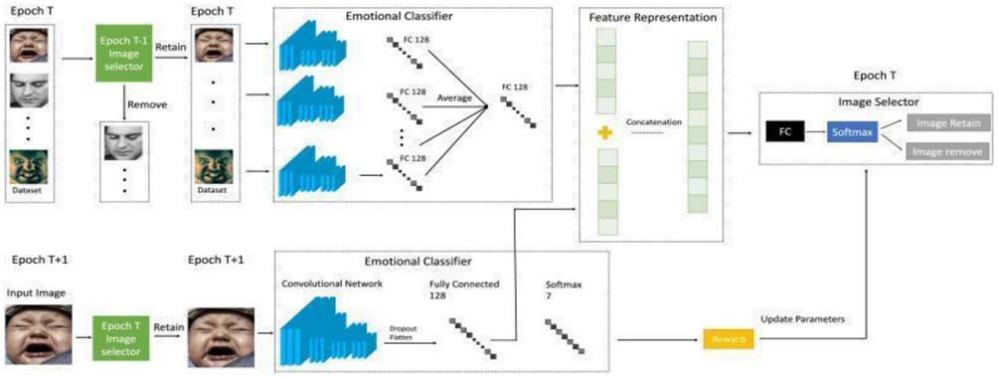
The application of reinforcement learning in complex emotion recognition systems.

In affective computing for complex emotion recognition, employing reinforcement learning (RL) offers significant advantages by addressing key challenges (Zhang et al., 2021). One major issue is handling unlabeled data, as complex emotions are often not explicitly labeled in datasets. RL, particularly through Deep Q-Networks (DQN), can learn from the environment via rewards and penalties, thus improving performance without the need for labeled data. Another challenge is identifying emotionally relevant intervals within data streams, such as video or physiological signals. RL-based segmentation dynamically learns to highlight these intervals, refining its strategy over time through rewards for accurate emotion recognition. It's also complex to integrate multimodal data, including facial expressions and physiological signals. RL excels in this by adaptively learning which signals are more indicative of specific emotions in varying contexts, enhancing the system's overall recognition capability. The system employs both facial expressions and physiological signals for emotion recognition. From facial expressions, it extracts confidence scores of seven basic emotions, valence-arousal (VA) levels, and 10 action units (AUs). For physiological signals, it utilizes remote photoplethysmography (rPPG) to derive heart rate (HR) and heart rate variability (HRV) indices, along with EEG and ECG signals (Wang et al., 2021). This combination of facial and physiological data provides a comprehensive approach to recognizing complex emotions. The variability and subtlety of complex emotions often reduce recognition accuracy with traditional methods. RL optimizes segmentation and decision making iteratively, focusing on the most informative data segments, and thus improving recognition accuracy. Additionally, emotional states change rapidly, posing a challenge for static models. RL's adaptive learning allows for real-time updates in understanding and segmentation, making the system robust and effective in capturing dynamic emotional states in real-world scenarios. By addressing these challenges, RL significantly enhances the performance and reliability of complex emotion recognition systems in affective computing. Another significant advantage of using RL for complex emotion recognition is its ability to learn from sparse and unlabeled data. By focusing on key segments with significant emotional information, the RL-based segmentation module ensures that the decision module receives the most relevant data, leading to better recognition accuracy (Mao et al., 2020). This method is particularly effective for complex emotions, which are often subtle and difficult to capture through simple observation. The RL approach allows the system to adapt and improve over time, refining its ability to detect and interpret complex emotional states. There is a step-by-step progression of emotions in interactions, which is comparable to how the action selected in reinforcement learning depends on the emotional state at that moment and the sequence of state transitions. The chosen activity, representing the target's recognition results and the current emotional state, also impacts the reward function. This reinforcement learning module is essential for identifying the target emotion using characteristics of the appropriate emotion pair. By effectively managing unlabeled data, identifying key emotional intervals, integrating multimodal data, and improving recognition accuracy through iterative optimization, RL-based systems offer a comprehensive solution to the complexities of emotion recognition (Malik et al., 2025; Sun et al., 2025; Zhang et al., 2025). The dynamic and adaptive nature of RL allows these systems to respond to rapid changes in emotional states, ensuring robust performance in real-world scenarios. The step-by-step progression and adaptation in RL mirror the evolving nature of human emotions, making it a powerful tool in affective computing. Reinforcement learning's ability to continuously improve through experience and feedback makes it particularly suited for the nuanced task of complex emotion recognition, providing a reliable and effective approach to understanding human emotions in various contexts, which is highlighted in Figure 11 (Alhagry et al., 2017; Moran and Gordon, 2023; Liu et al., 2024; Ramadan et al., 2024).

## 6 A review of related works

The review of related works in basic and complex emotion recognition systems reveals a spectrum of advancements, from foundational models focusing on principal emotions. In this section, these provided studies underscore the evolution toward more accurate and context-aware systems.

### 6.1 Basic emotion recognition

In this section, we aim to review some studies related to AI approaches corresponding to the recognition of emotion by considering facial expressions, ECG, and EEG signals. The construction of an artificial intelligence (AI) system that can recognize emotions from facial expressions. Jaiswal et al. (2020) developed an AI system for emotion detection using deep learning, specifically a convolutional neural network (CNN) architecture. This model focuses on three main processes: face detection, feature extraction, and emotion classification. CNN's ability to automatically extract features and classify emotions showcases significant accuracy, achieving 98.65% on the JAFFE dataset and 70.14% on the FERC-2013 dataset. Key advantages of this model include high accuracy, the efficacy of deep learning in reducing manual feature extraction, and scalability.

However, the model's limitations are its dependency on the quality and diversity of datasets, substantial computational requirements, and potential generalization issues across different populations and lighting conditions. Overall, while the CNN-based model marks a significant improvement over traditional methods, further enhancements are needed for broader application and real-world deployment. Lee et al. provided a novel multi-modal input approach that uses color, depth, and thermal recording videos to estimate dimensional emotion states was described (Lee et al., 2020). Based on attention-boosted feature volumes, the proposed networks, dubbed multi-modal recurrent attention networks (MRAN), robustly distinguish facial expressions by learning spatiotemporal attention volumes. Utilizing the depth and heat sequences as guide priors, a selective focus on emotional discriminative regions on the color sequence was applied. Additionally, a brand-new multi-modal facial expression recognition benchmark called multi-modal arousal-valence facial expression recognition (MAVFER) comprised continuous arousal-valence scores matching to films of color, depth, and thermal recording.

The outcomes of the experiments demonstrate that the approach was capable of producing state-of-the-art outcomes in color recording datasets for dimensional facial emotion recognition. Zhang et al. (2020) studied the implementation of several deep learning models, such as LSTM, convolutional neural networks (CNN), deep neural networks (DNN), and a hybrid model of CNN-LSTM, on the subject of their research of EEG-based emotion recognition. The popular DEAP dataset was applied for evaluation.

According to the experimental results, the CNN and CNN-LSTM models performed well in the categorization of EEG-based emotions, with accurate raw data extraction rates of 90.12% and 94.17%, respectively. Bazgir et al. (2018) researched on the DEAP dataset to build an AI system for the recognition of emotion based on EEG signals. Operating the discrete wavelet transform (DWT), EEG signals were divided into the gamma, beta, alpha, and theta frequency bands. Spectral features were then retrieved from each frequency band. To make the features mutually uncorrelated, principal component analysis (PCA) was used for the retrieved features while maintaining the same dimensionality as a transform. Emotional states were categorized by support vector machines (SVM), artificial neural networks (ANN), and k-nearest neighbors (KNN). With extracted features from ten EEG channels, the cross-validated SVM with radial basis function (RBF) kernel performs with 91.3% accuracy for arousal and 91.1% accuracy for valence in the beta frequency range. The purpose of the study (Sun and Lin, 2022) was to employ ECG signals to identify emotions. The data represented four different emotions: happy, thrilling, tranquil, and tense. A finite impulse filter is then used to de-noise the raw data. To improve the accuracy of emotion recognition, The Discrete Cosine Transform (DCT) to extract characteristics from the collected data was applied. Support Vector Machine (SVM), Random Forest, and K-NN classifiers are investigated. The optimal parameters for the SVM classifier are found using the Particle Swarm Optimization (PSO) approach.

The comparison of these classification techniques' findings shows that the SVM methodology recognizes emotions more accurately, which is useful in practical settings. Nguyen et al. (2018) provided a new transfer learning approach utilizing PathNet to explore knowledge accumulation within a dataset and transfer insights from one emotion dataset to enhance overall performance to solve the generalization problem of developed deep learning models, corresponding to a shortage of extensive emotion datasets. The proposed system by passing different series of investigations on SAVEE and eNTERFACE datasets enhances emotion recognition performance according to experimental results, outperforming recent state of-the-art methods that employ fine-tuning or pre-trained approaches. The highest recognition accuracy that the proposed system could obtain was 93.75% on SAVEE and 87.5% on eNTERFACE. Albraikan et al. (2018) studied on the E4, and MAHNOB datasets to boost the classifier's accuracy rate by utilizing peripheral physiological signals. A hybrid sensor fusion method based on a stacking model was presented, which enabled the simultaneous embedding of data from several sensors and emotion models within a model that was independent of the user.

As a fundamental model for classifying emotions, WMD-DTW, a weighted multidimensional DTW, and the k-nearest neighbors algorithm were employed. On top of the two base models, a high-level classifier was learned using the ensemble methods. Applying a meta-learning methodology, were able to demonstrate the ensemble method performs more effectively than any particular method. The result showed recognizing valence and arousal emotions achieved 94.0% and 93.6% employing the MAHNOB dataset. Table 4 provides an extensive overview of the techniques, results, advantages, and disadvantages of the featured study, along with additional pertinent research. It aims to assist in conducting a meticulous comparison in order to clarify the pros and cons associated with each study. In this way, it aims to enhance comprehension of the present status of research in the domain of emotion recognition using physical and physiological cues.

**Table 4:** Overview of mentioned studies and another studies on emotion recognition using physical and physiological cues.

**Study**	**Methodology**	**Datasets**	**Results (accuracy)**	**Advantages**	**Limitations**
Jaiswal et al. (2020)	CNN	JAFFE, FERC-2013	70.14% (FERC-2013), 98.65% (JAFFE)	High accuracy on JAFFE	Lower accuracy on FERC-2013
Lee et al. (2020)	MRAN	MAVFER	52.5%	Robust to multi-modal data	Computationally intensive
Zhang et al. (2020)	CNN-LSTM	DEAP	94.17%	High classification accuracy	Potential overfitting
Bazgir et al. (2018)	SVM	DEAP	91.3% (arousal), 91.1% (valence)	Effective feature extraction	Dependence on frequency bands
Sun and Lin (2022)	SVM	Four emotions (ECG signals)	90.5%	High practical accuracy	Limited emotion set
Nguyen et al. (2018)	PathNet	SAVEE, eNTERFACE	93.75% (SAVEE), 87.5% (eNTERFACE)	Improved generalization	Complexity in implementation
Albraikan et al. (2018)	Hybrid sensor fusion	E4, MAHNOB	94.0% (valence), 93.6% (arousal)	Effective multi-sensor integration	High computational cost
Lopes et al. (2017)	CNN	Custom dataset	94.3%	High accuracy, robust model	Limited to specific dataset
Tripathi et al. (2017)	CNN	DEAP	81.40% (valence), 73.36% (arousal)	Utilizes multiple neural networks	Limited to DEAP dataset

### 6.2 Complex emotion recognition

In this section, an overview of selected papers focusing on meta-learning approaches for the recognition of complex emotions based on basic emotions will be presented. This review is prompted by the limited existing research on complex emotions. The selected papers are categorized into three distinct groups: Continual learning and few-shot learning will be discussed in part 1. Part 2 will cover label noises, while the investigation of reinforcement learning will be undertaken in the final part.

#### 6.2.1 Part (1) continual learning and few-shot learning

Maiden and Nakisa (2023) presented a new method based on continual learning and few-shot learning that improves and maintains its understanding of basic expression classes to recognize new compound expression classes accurately with a few training samples. Data augmentation, knowledge extraction, and a revolutionary Predictive Sorting Memory Replay to prevent catastrophic forgetting and enhance performance with fewer training samples were used. A considerable association between the activations of features in basic expressions and those in compound expressions was discovered by comparing the Grad-CAM heat maps of images of basic expressions with those of compound expressions (Jayamohan and Yuvaraj, 2025). Continual learning outperforms non-continual learning methods in complicated face expression recognition, outperforming non-continual learning methods' state-of-the-art by 13.95%. The overall accuracy in new classes with 74.28% had demonstrated that continual learning for complex facial expression recognition played an essential role. This study is motivated by human cognition and learning patterns, and it is the first to use few-shot learning for complex facial expression identification, attaining the state-of-the-art with 100% accuracy while only requiring one training sample for each expression class.

To showcase significant improvements and practical applications in the domain of complex emotion recognition systems, Bhosale et al. (2022) proposed a few-shot adaptation method from EEG signals to address the lengthy calibration phase required by traditional Brain-Computer Interfaces (BCIs), hindering an optimal plug-and-play experience. Their model employed meta-learning to generalize well to new individuals with limited data, utilizing a few-shot learning framework trained on a small number of samples from previously unseen subjects, hence avoiding the need for retraining. Tested on the DEAP database with EEG recordings from 32 subjects watching music videos followed by emotion ratings, their method significantly improved emotion classification accuracy in terms of valence and arousal using only 20 reference samples from new subjects. Key contributions included quantifying the minimum calibration samples needed and introducing a 3-D convolutional recurrent embedding model to capture temporal relationships from spatially convolved EEG features. They explored various sampling strategies for support sets, finding that a combination of subject-dependent and subject-independent samples yielded competitive performance. In zero calibration scenarios, the model trained with subject-independent samples outperformed the supervised baseline. This system reduced the calibration burden and enhanced classification accuracy, advancing cross-subject EEG emotion recognition models and paving the way for more user-friendly and effective BCI applications.

By improving accuracy and robustness in recognizing micro-expressions for complex emotion recognition systems, a dual-branch meta-auxiliary learning method called LightmanNet for micro-expression recognition (MER) to address the challenges of limited data, subtle features, and individual differences in emotion detection was conducted by Wang et al. (2024). LightmanNet utilizes a bi-level optimization process: in the first level, it learns task-specific MER knowledge through two branches. The primary branch focuses on learning MER features via primary MER tasks. In contrast, the auxiliary branch guides the model by aligning micro-expressions with macro-expressions, leveraging their spatial and temporal similarities. This dual-branch approach ensures the model learns meaningful features and avoids noise. In the second level, the model refines the task-specific knowledge, enhancing its generalization and efficiency. The method enables the rapid acquisition of discriminative and generalizable MER knowledge from limited data. Extensive experiments demonstrated that LightmanNet significantly outperformed traditional, deep-learning, and meta-learning-based MER methods. The key contributions include addressing the data-level, feature-level, and decision-making-level challenges in MER, proposing a novel dual-branch meta-auxiliary learning method to improve model generalization and efficiency, and demonstrating its superior robustness and effectiveness. This work advances the field of complex emotion recognition systems by improving accuracy and robustness in recognizing micro-expressions.

#### 6.2.2 Part (2) label noise

In the study by Parrott (2001), Self-cure relation networks (SCRNet), a metric-based few-shot model that is resistant to label noise and capable of classifying facial images of new classes of emotions by only a few examples from each, was introduced as a solution to the complex emotion recognition via facial expressions problem that is demonstrated in a few-shot learning problem. By generating relation scores between the query image and the sparse samples of each new class, SCRNet establishes a distance metric based on deep information abstracted by convolutional neural networks and predicts an emotion category for a query image. Six basic emotion categories such as Happiness, Surprise, Sadness, Fear, Disgust, and Anger for facial expression detection to more intricate and compound emotions had developed. Given the difficulty in obtaining large datasets and the high level of expertise required for sophisticated facial expression interpretation, SCRNet addresses the label noise issue. It does so by using a class prototype, which is maintained in external memory during the meta-training phase, to assign corrected labels to noisy data. The effectiveness of the proposed approach has been verified on both synthetic noise datasets and public datasets.

#### 6.2.3 Part (3) reinforcement learning

A bionic two-system architecture for recognizing complicated emotions was proposed by Wu et al. (2023). The design resembles how the human brain responds to challenges and makes decisions. A quick compound sensing module is System I. System II is a slower cognitive decision-making component that interacts with data processing more. System I has two branches: one for physiological measurement, which is a practical image-only implementation, and one for facial expression feature representation, comprising fundamental emotion, action units, and valence arousal detection. In System II, a decision module with segmentation is used to verify that the chosen time includes the occurrence of the emotion and to iteratively optimize the emotion information in a particular segment via reinforcement learning. By achieving an accuracy of 94.15% for basic emotion recognition on the BP4D database with five classes and an accuracy of 68.75% for binary valence arousal classification on the DEAP, the recommended approach outperforms advanced emotion recognition tasks. The recognition accuracy on both databases exceeds 70% for a selection of complicated emotions, which is a massive improvement.

## 7 Discussion

In this study, we reviewed research papers to provide an overview of current research on meta-learning approaches, focusing on three types of unstructured data, including facial expressions, EEG, and ECG signals. Considering recent developments, the domain of complex emotion systems is still in its early stages, with very few papers examining meta-learning applications in these three domains.

An important insight from this review is that the limited quality and scope of current datasets remain a significant barrier to progress in complex emotion recognition. Most publicly available datasets do not contain the contextual richness, multi stage emotional transitions, or interactive scenarios that give rise to higher level emotions. The lack of jointly recorded multimodal signals, such as EEG ECG facial dynamics and body gestures, further reduces the ecological validity of existing resources. As a result, evaluating CERS models becomes difficult and meta-learning techniques cannot benefit from diverse and well-structured tasks. Advancing the field requires the development of large-scale multimodal datasets that capture naturally occurring compound emotions with detailed annotations, diverse participants, and high temporal resolution.

In complex emotion recognition systems, facial expressions, along with EEG and ECG signals, play a vital role. The preprocessing steps for facial expression data are similar to signal processing techniques, but are tailored for image data. Standardizing input size and removing irrelevant background information through resizing and cropping facial images helps expedite processing and reduce the computational burden. Additionally, normalization techniques ensure consistent pixel intensities across images, improving model performance by mitigating potential biases. Data augmentation methods like rotation, flipping, and adding noise to facial images enhance dataset diversity and prevent overfitting, similar to signal processing counterparts. Implementing these preprocessing steps on facial expression data helps extract more meaningful features, leading to accurate and robust emotion classification. Employing facial expression data, EEG, and ECG signals in a complex emotion recognition system delivers a holistic view of an individual's emotional state. This strategy utilizes supplementary data from various modalities to improve overall performance and dependability in real-world scenarios.

### 7.1 Evaluation metrics

**Figure 12:**
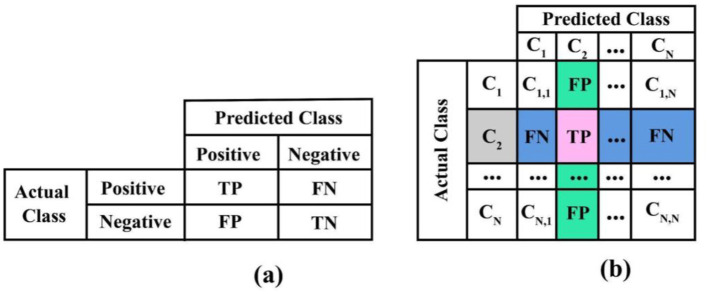
**(a, b)** Confusion matrix based on the Complex Emotion Recognition System (CERS) for multi-class emotion classification. Colored cells highlight: TP (pink on diagonal), FP (green), FN (blue); diagonal cells represent correct predictions, while off-diagonal cells indicate misclassifications (including FP and FN for each class pair).

The development of CERS necessitates a robust evaluation framework tailored to the unique challenges of emotion recognition, where all proposed systems must experience evaluation on standardized datasets, comparing predicted emotion ratings or labels with ground truth. In classification problems, classifiers are frequently evaluated using a confusion matrix-based technique, as shown in Figure 12 (Ko, 2018; Li et al., 2022).

This approach provides five critical classification metrics for performance comparison; as a consequence, these criteria for the evaluation of models, including precision, recall, accuracy, specificity, and F1-score, have been constructed. Precision demonstrates the proportion of accurately detected instances of a certain emotion, emphasizing the system's ability to identify emotional states. In contrast, recall evaluates the ability of the system to detect all instances of a specific emotion in the dataset, assuring sensitivity to emotional expressions. Accuracy provides a wide overview of categorization correctness, considering true positives, true negatives, false positives, and false negatives. Specificity, represented by the True Negative (TN) rate, assesses the system's accuracy in correctly identifying instances devoid of specific emotions, crucial for distinguishing genuine emotional expressions from non-emotional states. However, in instances with unbalanced data, accuracy alone may not be sufficient, emphasizing the significance of prioritizing recall and precision for a nuanced assessment. By emphasizing recall and precision, CERS developers can better evaluate system performance, particularly in handling imbalanced datasets, leading to continuous refinement and optimization efforts in the pursuit of more accurate and reliable emotion recognition systems (Ackermann et al., 2016; Russo et al., 2018; Ramírez-Arias et al., 2022). The Equations 1–5 are employed to calculate each metric, providing a comprehensive evaluation of the CERS performance.


$$\begin{array}{l} \text{Accuracy}=\;\frac{\text{TP }+\text{ TN}}{\text{TP }+\text{ FN }+\text{ TN}+\text{FP}} \end{array}$$ (1)

$$\begin{array}{l} \text{Precision}=\;\frac{\text{TP}}{\text{TP }+\text{ FP}} \end{array}$$ (2)

$$\begin{array}{l} Recall=\frac{\text{TP}}{\text{TP }+\text{ FN }} \end{array}$$ (3)

$$\begin{array}{l} Specificity=\frac{\text{TN }}{\text{TN }+\text{ FP}} \end{array}$$ (4)

$$\begin{array}{l} F1-score=\frac{2\;.\text{Precision}.\text{recall }}{\text{Precision }+\text{ recall}} \end{array}$$ (5)

To enable a direct comparison among different approaches, all methods were evaluated on various datasets. Table 5 summarizes the results obtained for several representative models and the proposed hybrid approach, providing the dataset name, Participants, Emotion Classes, Emotion Type, Duration or Samples, method, modality, Accuracy, F1 Score, and result achieved for emotion recognition. It is important to emphasize that metrics such as precision, recall, and specificity were not reported in the reviewed studies, and therefore, only the performance measures provided by the original authors are included in the table.

**Table 5:** Summary and comparison of different methods for emotion recognition.

**Reference**	**Dataset name**	**Participants**	**Emotion classes**	**Emotion type**	**Modality**	**Duration or samples**	**Method**	**Accuracy**	**F1 score**
Zhao and Xu (2019)	CMED	26 to 32 subjects across source datasets	6 basic plus 11 compound	Complex and basic	EEG unimodal	Micro expression sequences	Hierarchical recurrent neural network	78.33%	Not reported
Takalkar and Xu (2017)	CASME	19 subjects	8 classes	Complex and basic	Facial images and video	180 video samples	LBP TOP	90.40%	89.80%
Mavani et al. (2017)	CFEE	230 subjects	21 classes	Complex and basic	Facial images	5,060 labeled images	Deep CNN multi task	81.50%	Not reported
Han et al. (2023)	FER- 2013	35,887 images no subject ID	7 classes	Basic	Facial images	35 k images	Attention based Bilateral Feature Fusion Network	91.13%	90.10%
Yan et al. (2022)	RAF- DB	More than 15 k subjects online sourced	7 basic and 12 compound	Complex	Facial images	29,672 images	Self supervised label denoising	90.87%	90.50%
Benda and Scherf (2020)	CEED	8 actors and 800 raters	15 classes (6 basic and 9 complex)	Complex	EEG plus peripheral physiology plus facial images	480 images	SVM with statistical features	68.20%	Not reported

Acc, Accuracy; Pre, Precision; Rec, Recall; Spe, Specificit y; F1, F1-score.

Based on the results presented in Table 4, various methods for complex emotion recognition across different datasets and modalities have been compared. CMED (Zhao and Xu, 2019) achieved an accuracy of 78.33% using Hierarchical Recurrent Neural Networks (HRNN) with EEG data, though other metrics such as precision, recall, specificity, and F1-score were not reported. In contrast, CASME (Takalkar and Xu, 2017), which utilized the LBP-TOP method with facial expressions (images/videos), achieved a higher accuracy of 90.4%, with an F1-score of 89.8%, demonstrating the effectiveness of facial expression-based approaches in recognizing subtle micro-expressions. CFEE (Mavani et al., 2017), applying a Deep CNN with multi-task learning, achieved an accuracy of 81.5%, but no other metrics were provided. FER-2013 (Han et al., 2023) achieved the highest accuracy at 91.13% using an Attention-based Bilateral Feature Fusion Network (ABFFN), with a reported F1-score of 90.1%, highlighting the potential of attention mechanisms in focusing on relevant facial features for emotion recognition. RAF-DB (Yan et al., 2022) also achieved high accuracy (90.87%) using Self-Supervised Label Denoising (SSLD) with facial expression data, and an F1-score of 90.5%, demonstrating that improving label quality can significantly enhance model performance. On the other hand, CEED (Benda and Scherf, 2020), which used SVM with statistical features on multimodal data (EEG and peripheral physiology), showed the lowest accuracy at 68.2%, with no additional metrics reported. Despite its lower performance, CEED's multimodal approach provides a richer dataset for recognizing complex emotions, though it highlights the challenges in combining multiple data sources. Overall, facial expression-based methods generally yielded higher accuracy, with FER-2013 performing the best. At the same time, multimodal approaches like CEED and CMED faced greater challenges but still showed promising results in complex emotion recognition.

## 8 Open research challenges

In the domain of complex recognition systems, several open research challenges continue to limit model robustness, scalability, and real-world applicability. These challenges span technical, methodological, and ethical and practical dimensions, and they directly reflect the limitations identified throughout this review, particularly those related to dataset scarcity, variability in recording protocols, and limited explainability as described by Zhu et al. (2025). From a technical perspective, key difficulties include generalization across subjects and domains, inconsistencies in multimodal fusion, and the vulnerability of recognition systems to label noise and distribution shifts. As highlighted in Sections 4–6, current deep learning and meta-learning models remain highly sensitive to heterogeneous datasets, sensor artifacts, and demographic imbalance. Although few-shot and continual learning approaches offer promising mechanisms for reducing calibration, time as suggested by Khoee et al. (2024), their scalability to large and diverse datasets remains limited. To address this gap, more robust task-aware meta-learning pipelines, noise-resistant training strategies, and adaptive fusion mechanisms that can operate under missing or asynchronous modalities are required, as emphasized by Zheng et al. (2025) and Wang et al. (2025).

From a methodological standpoint, the absence of standardized preprocessing protocols, unified evaluation metrics, and reproducible experimental setups continues to undermine scientific progress. The inconsistency of annotation standards, sampling rates, and biosignal processing pipelines limits the comparability of existing models and prevents reliable benchmarking. As the findings summarized in Section 5 indicate, the development and adoption of community-shared preprocessing frameworks and comprehensive reporting practices are essential for improving reliability and supporting consistent model assessment (Jedličková, 2025).

Equally important are the ethical and practical challenges associated with privacy risks, demographic bias, limited transparency, and the broader societal impact of automated decisions. Because biosignal-based recognition systems inherently process sensitive personal information, issues related to data security, fairness, and interpretability remain central concerns, as highlighted by Dhinakaran et al. (2025) and Poszler et al. (2025). The discussions from Sections 3 and 6 further demonstrate that recognition systems deployed in real-world contexts such as mental health support, education, and human–machine interaction require transparent explanations, bias-aware representations, and privacy-preserving learning approaches. Advances in explainable artificial intelligence, fairness-aware optimization, and inclusive data collection practices therefore represent urgent research directions, as supported by Mourid et al. (2025) and Ravi et al. (2025).

Considering these interconnected challenges, a set of research priorities can be outlined. In the short term, the focus should be on establishing standardized preprocessing and evaluation pipelines, developing more robust label-noise handling strategies, and incorporating interpretable modules into recognition systems. In the mid-term, research should prioritize the creation of balanced multimodal datasets, the design of adaptive fusion models capable of operating under missing-modality conditions, and the improvement of generalizable meta-learning frameworks through stronger cross-domain adaptation. In the long term, the field should aim toward the development of privacy-preserving, energy-efficient, and ethically aligned recognition systems that can offer real-time self-calibration, transparent decision-making, and sustainable deployment across a variety of real-world environments.

Following these priorities, a coherent research roadmap emerges. The field should first strengthen its foundations by improving standardization, robustness to noise, and baseline interpretability. It should then advance toward scalable multimodal and meta-learning systems that can cope with the diversity and complexity of real-world conditions. Ultimately, the objective is to achieve a mature generation of trustworthy, privacy-conscious, and socially responsible recognition systems. By linking these challenges and future directions to the limitations and findings presented throughout this review, this roadmap provides a structured and evidence-driven vision for the advancement of complex recognition systems.

## 9 Conclusions

This study provides a comprehensive review of CERS and its role in emotion recognition within complex tasks. Unlike traditional methods focused on basic emotion recognition through facial expressions or physiological signals like EEG and ECG, our review highlights the growing potential of CERS in capturing emotions in more intricate contexts, with applications across healthcare, education, and human-computer interaction. Our findings demonstrate that CERS, even in its early stages, shows promise in identifying emotions tied to complex tasks, offering significant potential for enhancing human-computer interaction. The integration of multi-modal sensor data further strengthens its capacity to accurately infer specific emotions, paving the way for more personalized and intelligent systems. This review also lays the groundwork for future investigations into the broader use of emotion recognition technologies, particularly in dynamic, real-world settings.

Future research should consider broader advancements in emotion recognition systems, focusing on improving multi-modal data fusion and real-time adaptation to dynamic environments. Investigating new approaches in meta-learning, such as continual learning or few-shot learning, could help these systems better adapt to various user contexts. Additionally, ethical considerations must remain a core focus, especially concerning the societal implications of CERS deployment. Ensuring fairness, transparency, and the responsible use of emotion recognition technologies will be crucial for their ethical and sustainable development.

## References

[B1] Ackermann P. Kohlschein C. Bitsch J. A. Wehrle K. Jeschke S. (2016). “EEG-based automatic emotion recognition: feature extraction, selection and classification methods,” in 2016 IEEE 18th International Conference on e-Health Networking, Applications and Services (Healthcom) (Piscataway, NJ: IEEE), 1–6.

[B2] Adolphs R. (2017). How should neuroscience study emotions? By distinguishing emotion states, concepts, and experiences. Soc. Cogn. Affect. Neurosci. 12, 24–31. doi: 10.1093/scan/nsw153 27798256 PMC5390692

[B3] Ahmed N. Al Aghbari Z. Girija S. (2023). A systematic survey on multimodal emotion recognition using learning algorithms. Intell. Syst. Appl. 17:200171. doi: 10.1016/j.iswa.2022.200171

[B4] Albraikan A. Tobón D. P. El Saddik A. (2018). Toward user-independent emotion recognition using physiological signals. IEEE Sens. J. 19, 8402–8412. doi: 10.1109/JSEN.2018.2867221

[B5] Alhagry S. Fahmy A. A. El-Khoribi R. A. (2017). Emotion recognition based on EEG using LSTM recurrent neural network. Int. J. Adv. Comput. Sci. Appl. 8, 355–358. doi: 10.14569/IJACSA.2017.081046

[B6] Bazgir O. Mohammadi Z. Habibi S. a. H. (2018). “Emotion recognition with machine learning using EEG signals,” in 2018 25th National and 3rd International Iranian Conference on Biomedical Engineering (ICBME) (Piscataway, NJ: IEEE), 1–5.

[B7] Beall A. T. Tracy J. L. (2017). Emotivational psychology: how distinct emotions facilitate fundamental motives. Soc. Personal. Psychol. Compass 11:e12303. doi: 10.1111/spc3.12303

[B8] Benda M. S. Scherf K. S. (2020). The Complex Emotion Expression Database: a validated stimulus set of trained actors. PLoS ONE 15:e0228248. doi: 10.1371/journal.pone.0228248 32012179 PMC6996812

[B9] Bhosale S. Chakraborty R. Kopparapu S. K. (2022). Calibration free meta learning based approach for subject independent EEG emotion recognition. Biomed. Signal Process. Control 72:103289. doi: 10.1016/j.bspc.2021.103289

[B10] Black M. H. Chen N. T. Lipp O. V. Bölte S. Girdler S. (2020). Complex facial emotion recognition and atypical gaze patterns in autistic adults. Autism 24, 258–262. doi: 10.1177/1362361319856969 31216863

[B11] Booth B. M. Mundnich K. Feng T. Nadarajan A. Falk T. H. Villatte J. L. . (2019). Multimodal human and environmental sensing for longitudinal behavioral studies in naturalistic settings: framework for sensor selection, deployment, and management. J. Med. Internet Res. 21:e12832. doi: 10.2196/12832 31432781 PMC6719486

[B12] Calvo R. A. D'mello S. (2010). Affect detection: an interdisciplinary review of models, methods, and their applications. IEEE Trans. Affect. Comput. 1, 18–37. doi: 10.1109/T-AFFC.2010.1

[B13] Chaidi I. Drigas A. (2020). Autism, expression, and understanding of emotions: literature review. Int. J. Online Biomed. Eng. 16, 94–112. doi: 10.3991/ijoe.v16i02.11991

[B14] Chang Y. Li W. Peng J. Tang B. Kang Y. Lei Y. . (2021). Reviewing continual learning from the perspective of human-level intelligence. arXiv [Preprint]. arXiv:2111.11964. doi: 10.48550/arXiv.2111.11964

[B15] Chaubey M. S. Pathrotkar M. N. (2023). Facial recognition AI: a powerful tool for emotion detection and characterization. J. Data Acquis. Process. 38:1914.

[B16] Chen H. Shi H. Liu X. Li X. Zhao G. (2023). SMG: a micro-gesture dataset towards spontaneous body gestures for emotional stress state analysis. Int. J. Comput. Vis. 131, 1346–1366. doi: 10.1007/s11263-023-01761-6

[B17] Chen Z. S. Galatzer-Levy I. R. Bigio B. Nasca C. Zhang Y. (2022). Modern views of machine learning for precision psychiatry. Patterns 3, 1–37. doi: 10.1016/j.patter.2022.100602 36419447 PMC9676543

[B18] Chiang K.-W. Tan C.-H. Hong W.-P. Yu R.-L. (2024). Disgust-specific impairment of facial emotion recognition in Parkinson's disease patients with mild cognitive impairment. Soc. Cogn. Affect. Neurosci. 19:nsae073. doi: 10.1093/scan/nsae073 39417289 PMC11561469

[B19] Chuang Y.-H. Tan C.-H. Su H.-C. Chien C.-Y. Sung P.-S. Lee T.-L. . (2022). Hypomimia may influence the facial emotion recognition ability in patients with Parkinson's disease. J. Parkinson's Dis. 12, 185–197. doi: 10.3233/JPD-212830 34569974

[B20] Coskun A. (2019). Design for long-term tracking: insights from a six-month field study exploring users' experiences with activity trackers. Des. J. 22, 665–686. doi: 10.1080/14606925.2019.1634447

[B21] Côté-Allard U. Campbell E. Phinyomark A. Laviolette F. Gosselin B. Scheme E. (2020). Interpreting deep learning features for myoelectric control: a comparison with handcrafted features. Front. Bioeng. Biotechnol. 8:158. doi: 10.3389/fbioe.2020.00158 32195238 PMC7063031

[B22] Cui K. Li J. Liu Y. Zhang X. Hu Z. Wang M. (2025). PhysioSync: temporal and cross-modal contrastive learning inspired by physiological synchronization for EEG-based emotion recognition. arXiv [Preprint]. arXiv:2504.17163. doi: 10.1109/TCSS.2025.3602913

[B23] Davison A. K. Lansley C. Costen N. Tan K. Yap M. H. (2016). SAMM: a spontaneous micro-facial movement dataset. IEEE Trans. Affect. Comput. 9, 116–129. doi: 10.1109/TAFFC.2016.2573832

[B24] Dhinakaran D. Raja S. E. Jasmine J. J. Kumar P. V. Ramani R. (2025). “The future of well-being: AI-powered health management with privacy at its core,” in Wellness Management Powered by AI Technologies (Beverly, MA: Scrivener Publishing), 363–402.

[B25] Ding Y. Tian X. Yin L. Chen X. Liu S. Yang B. . (2019). “Multi-scale relation network for few-shot learning based on meta-learning,” in International Conference on Computer Vision Systems (Berlin: Springer), 343–352.

[B26] Do N.-T. Kim S.-H. Yang H.-J. Lee G.-S. Yeom S. (2021). Context-aware emotion recognition in the wild using spatio-temporal and temporal-pyramid models. Sensors 21:2344. doi: 10.3390/s21072344 33801739 PMC8036494

[B27] Dora C. Biswal P. K. (2020). Engineering approaches for ECG artefact removal from EEG: a review. Int. J. Biomed. Eng. Technol. 32, 351–383. doi: 10.1504/IJBET.2020.107203

[B28] Du S. Tao Y. Martinez A. M. (2014). Compound facial expressions of emotion. Proc. Natl. Acad. Sci. U.S.A. 111, E1454–E1462. doi: 10.1073/pnas.1322355111 24706770 PMC3992629

[B29] Du Y. Li P. Cheng L. Zhang X. Li M. Li F. (2024). Attention-based 3D convolutional recurrent neural network model for multimodal emotion recognition. Front. Neurosci. 17:1330077. doi: 10.3389/fnins.2023.1330077 38268710 PMC10805863

[B30] Ekman P. (1992). An argument for basic emotions. Cogn. Emot. 6, 169–200. doi: 10.1080/02699939208411068

[B31] Ekman P. Cordaro D. (2011). What is meant by calling emotions basic. Emot. Rev. 3, 364–370. doi: 10.1177/1754073911410740

[B32] Erat K. Sahin E. B. Dogan F. Merdanoglu N. Akcakaya A. Durdu P. O. (2024). Emotion recognition with EEG-based brain-computer interfaces: a systematic literature review. Multimedia Tools Appl. 83, 79647–79694. doi: 10.1007/s11042-024-18259-z

[B33] Fabiano D. Canavan S. (2019). “Emotion recognition using fused physiological signals,” in 2019 8th International Conference on Affective Computing and Intelligent Interaction (ACII) (Piscataway, NJ: IEEE), 42–48.

[B34] Fan T. Qiu S. Wang Z. Zhao H. Jiang J. Wang Y. . (2023). A new deep convolutional neural network incorporating attentional mechanisms for ECG emotion recognition. Comput. Biol. Med. 159:106938. doi: 10.1016/j.compbiomed.2023.106938 37119553

[B35] Fdez J. Guttenberg N. Witkowski O. Pasquali A. (2021). Cross-subject EEG-based emotion recognition through neural networks with stratified normalization. Front. Neurosci. 15:626277. doi: 10.3389/fnins.2021.626277 33613187 PMC7888301

[B36] Feng Y. Chen J. Xie J. Zhang T. Lv H. Pan T. (2022). Meta-learning as a promising approach for few-shot cross-domain fault diagnosis: algorithms, applications, and prospects. Knowl. Based Syst. 235:107646. doi: 10.1016/j.knosys.2021.107646

[B37] Gandhi B. Saxena S. Jain P. (2023). “Emotion recognition: a review,” in Microelectronics, Circuits and Systems: Select Proceedings of Micro2021 (Singapore: Springer Nature Singapore Pte Ltd.), 371–379.

[B38] Gandhi R. K. (2022). Performance Analysis of Meta-learning and Contrastive Learning for Speech Emotion Recognition (Master's thesis). Eindhoven University of Technology. Available online at: https://research.tue.nl/en/studentTheses/performance-analysis-of-meta-learning-and-contrastive-learning-fo (Accessed June 29, 2022).

[B39] Giannopoulos P. Perikos I. Hatzilygeroudis I. (2017). “Deep learning approaches for facial emotion recognition: a case study on FER-2013,” in Advances in Hybridization of Intelligent Methods: Models, Systems and Applications (Berlin: Springer), 1–16.

[B40] Grandjean D. Sander D. Scherer K. R. (2008). Conscious emotional experience emerges as a function of multilevel, appraisal-driven response synchronization. Conscious. Cogn. 17, 484–495. doi: 10.1016/j.concog.2008.03.019 18448361

[B41] Greco A. Strisciuglio N. Vento M. Vigilante V. (2023). Benchmarking deep networks for facial emotion recognition in the wild. Multimedia Tools Appl. 82, 11189–11220. doi: 10.1007/s11042-022-12790-7

[B42] Grilo Jr M. Moraes C. P. Coelho B. F. O. Massaranduba A. B. R. Fantinato D. Ramos R. P. . (2023). Artifact removal for emotion recognition using mutual information and Epanechnikov kernel. Biomed. Signal Process. Control 83:104677. doi: 10.1016/j.bspc.2023.104677

[B43] Gu S. Wang F. Patel N. P. Bourgeois J. A. Huang J. H. (2019). A model for basic emotions using observations of behavior in Drosophila. Front. Psychol. 10:781. doi: 10.3389/fpsyg.2019.00781 31068849 PMC6491740

[B44] Guerdelli H. Ferrari C. Barhoumi W. Ghazouani H. Berretti S. (2022). Macro-and micro-expressions facial datasets: a survey. Sensors 22:1524. doi: 10.3390/s22041524 35214430 PMC8879817

[B45] Güntekin B. Hanoglu L. Aktürk T. Fide E. Emek-Savaş D. D. Ruşen E. . (2019). Impairment in recognition of emotional facial expressions in Alzheimer's disease is represented by EEG theta and alpha responses. Psychophysiology 56:e13434. doi: 10.1111/psyp.13434 31264726

[B46] Guo J. Lei Z. Wan J. Avots E. Hajarolasvadi N. Knyazev B. . (2018). Dominant and complementary emotion recognition from still images of faces. IEEE Access 6, 26391–26403. doi: 10.1109/ACCESS.2018.2831927

[B47] Han B. Kim H. Kim G. J. Hwang J.-I. (2023). “Masked FER-2013: augmented dataset for facial expression recognition,” in 2023 IEEE Conference on Virtual Reality and 3D User Interfaces Abstracts and Workshops (VRW) (Piscataway, NJ: IEEE), 747–748.

[B48] Hargrave R. Maddock R. J. Stone V. (2002). Impaired recognition of facial expressions of emotion in Alzheimer's disease. J. Neuropsychiatry Clin. Neurosci. 14, 64–71. doi: 10.1176/jnp.14.1.64 11884657

[B49] Hassouneh A. Mutawa A. Murugappan M. (2020). Development of a real-time emotion recognition system using facial expressions and EEG based on machine learning and deep neural network methods. Informatics Med. Unlocked 20:100372. doi: 10.1016/j.imu.2020.100372

[B50] He X. Sygnowski J. Galashov A. Rusu A. A. Teh Y. W. Pascanu R. (2019). Task agnostic continual learning via meta learning. arXiv [Preprint]. arXiv:1906.05201. doi: 10.48550/arXiv.1906.05201

[B51] Huang Y. Chen F. Lv S. Wang X. (2019). Facial expression recognition: a survey. Symmetry 11:1189. doi: 10.3390/sym11101189

[B52] Islam M. R. Moni M. A. Islam M. M. Rashed-Al-Mahfuz M. Islam M. S. Hasan M. K. . (2021). Emotion recognition from EEG signal focusing on deep learning and shallow learning techniques. IEEE Access 9, 94601–94624. doi: 10.1109/ACCESS.2021.3091487

[B53] Izard C. E. (2007). Basic emotions, natural kinds, emotion schemas, and a new paradigm. Perspect. Psychol. Sci. 2, 260–280. doi: 10.1111/j.1745-6916.2007.00044.x 26151969

[B54] Jaiswal A. Raju A. K. Deb S. (2020). “Facial emotion detection using deep learning,” in 2020 International Conference for Emerging Technology (INCET) (Piscataway, NJ: IEEE), 1–5.

[B55] Jayamohan M. Yuvaraj S. (2025). A novel human action recognition using Grad-CAM visualization with gated recurrent units. Neural Comput. Appl. 37, 10835–10850. doi: 10.1007/s00521-025-10978-0

[B56] Jedličková A. (2025). Ethical approaches in designing autonomous and intelligent systems: a comprehensive survey towards responsible development. AI Soc. 40, 2703–2716. doi: 10.1007/s00146-024-02040-9

[B57] Joloudari J. H. Maftoun M. Nakisa B. Alizadehsani R. Yadollahzadeh-Tabari M. (2024). Complex emotion recognition system using basic emotions via facial expression, EEG, and ECG signals: a review. arXiv [Preprint]. arXiv:2409.07493. doi: 10.48550/arXiv.2409.07493 PMC12989340 41847068

[B58] Kamble K. Sengupta J. (2023). A comprehensive survey on emotion recognition based on electroencephalograph (EEG) signals. Multimedia Tools Appl. 82, 27269–27304. doi: 10.1007/s11042-023-14489-9

[B59] Kang H. Lee M. (2025). “Multimodal emotion recognition from EEG and ECG signals via parallel fusion of graph convolution and LSTMs,” in 2025 47th Annual International Conference of the IEEE Engineering in Medicine and Biology Society (EMBC) (Piscataway, NJ: IEEE), 1–6. 10.1109/EMBC58623.2025.11253201 41335639

[B60] Kanjo E. Younis E. M. Ang C. S. (2019). Deep learning analysis of mobile physiological, environmental and location sensor data for emotion detection. Inf. Fusion 49, 46–56. doi: 10.1016/j.inffus.2018.09.001

[B61] Karimi D. Dou H. Warfield S. K. Gholipour A. (2020). Deep learning with noisy labels: exploring techniques and remedies in medical image analysis. Med. Image Anal. 65:101759. doi: 10.1016/j.media.2020.101759 32623277 PMC7484266

[B62] Keltner D. Buswell B. N. (1997). Embarrassment: its distinct form and appeasement functions. Psychol. Bull. 122:250. doi: 10.1037/0033-2909.122.3.250 9354148

[B63] Khare S. K. Blanes-Vidal V. Nadimi E. S. Acharya U. R. (2024). Emotion recognition and artificial intelligence: a systematic review (2014–2023) and research recommendations. Inf. Fusion 102:102019. doi: 10.1016/j.inffus.2023.102019

[B64] Khateeb M. Anwar S. M. Alnowami M. (2021). Multi-domain feature fusion for emotion classification using DEAP dataset. IEEE Access 9, 12134–12142. doi: 10.1109/ACCESS.2021.3051281

[B65] Khoee A. G. Yu Y. Feldt R. (2024). Domain generalization through meta-learning: a survey. Artif. Intell. Rev. 57:285. doi: 10.1007/s10462-024-10922-z

[B66] Kim C.-L. Kim B.-G. (2023). Few-shot learning for facial expression recognition: a comprehensive survey. J. Real-Time Image Process. 20:52. doi: 10.1007/s11554-023-01310-x

[B67] Kim C.-M. Hong E. J. Chung K. Park R. C. (2020). Driver facial expression analysis using LFA-CRNN-based feature extraction for health-risk decisions. Appl. Sci. 10:2956. doi: 10.3390/app10082956

[B68] Ko B. C. (2018). A brief review of facial emotion recognition based on visual information. Sensors 18:401. doi: 10.3390/s18020401 29385749 PMC5856145

[B69] Koelstra S. Muhl C. Soleymani M. Lee J.-S. Yazdani A. Ebrahimi T. . (2011). DEAP: a database for emotion analysis; using physiological signals. IEEE Trans. Affect. Comput. 3, 18–31. doi: 10.1109/T-AFFC.2011.15

[B70] Kusuma G. P. Jonathan J. Lim A. (2020). Emotion recognition on FER-2013 face images using fine-tuned VGG-16. Adv. Sci. Technol. Eng. Syst. J. 5, 315–322. doi: 10.25046/aj050638

[B71] Lee J. Kim S. Kim S. Sohn K. (2020). Multi-modal recurrent attention networks for facial expression recognition. IEEE Trans. Image Process. 29, 6977–6991. doi: 10.1109/TIP.2020.2996086

[B72] Lesort T. Lomonaco V. Stoian A. Maltoni D. Filliat D. Díaz-Rodríguez N. (2020). Continual lifelong learning for robotics: definition, framework, learning strategies, opportunities and challenges. Inf. Fusion 58, 52–68. doi: 10.1016/j.inffus.2019.12.004

[B73] Li H. Xu H. (2020). Deep reinforcement learning for robust emotional classification in facial expression recognition. Knowl. Based Syst. 204:106172. doi: 10.1016/j.knosys.2020.106172

[B74] Li J. Wang Y. See J. Liu W. (2019). Micro-expression recognition based on 3D flow convolutional neural network. Pattern Anal. Appl. 22, 1331–1339. doi: 10.1007/s10044-018-0757-5

[B75] Li L. Chen W. (2025). Cross-modal alignment and fusion of EEG-visual based on mixed attention mechanism for emotion recognition. Cogn. Neurodyn. 19, 1–20. doi: 10.1007/s11571-025-10372-5 41215980 PMC12596239

[B76] Li L. Mu X. Li S. Peng H. (2020). A review of face recognition technology. IEEE Access 8, 139110–139120. doi: 10.1109/ACCESS.2020.3011028

[B77] Li S. Deng W. (2020). Deep facial expression recognition: a survey. IEEE Trans. Affect. Comput. 13, 1195–1215. doi: 10.1109/TAFFC.2020.2981446

[B78] Li X. Zhang Y. Tiwari P. Song D. Hu B. Yang M. . (2022). EEG based emotion recognition: a tutorial and review. ACM Comput. Surv. 55, 1–57. doi: 10.1145/3524499

[B79] Liu H. Lou T. Zhang Y. Wu Y. Xiao Y. Jensen C. S. . (2024). EEG-based multimodal emotion recognition: a machine learning perspective. IEEE Trans. Instrum. Meas. 73, 1–29. doi: 10.1109/TIM.2024.3369130

[B80] Lopes A. T. De Aguiar E. De Souza A. F. Oliveira-Santos T. (2017). Facial expression recognition with convolutional neural networks: coping with few data and the training sample order. Pattern Recognit. 61, 610–628. doi: 10.1016/j.patcog.2016.07.026

[B81] Maiden A. Nakisa B. (2023). Complex facial expression recognition using deep knowledge distillation of basic features. arXiv [Preprint]. arXiv:2308.06197. doi: 10.48550/arXiv.2308.06197

[B82] Majhi B. Das N. Chakraborty M. (2024). “Analyzing emotional responses to audio-visual stimuli through heart rate variability analysis,” in 2024 IEEE International Students' Conference on Electrical, Electronics and Computer Science (SCEECS) (Piscataway, NJ: IEEE), 1–6.

[B83] Malik P. Singh J. Ali F. Sehra S. S. Kwak D. (2025). Action unit based micro-expression recognition framework for driver emotional state detection. Sci. Rep. 15:27824. doi: 10.1038/s41598-025-12245-7 40738929 PMC12311022

[B84] Mao Y. He Y. Liu L. Chen X. (2020). Disease classification based on eye movement features with decision tree and random forest. Front. Neurosci. 14:798. doi: 10.3389/fnins.2020.00798 32848569 PMC7423879

[B85] Mavani V. Raman S. Miyapuram K. P. (2017). “Facial expression recognition using visual saliency and deep learning,” in Proceedings of the IEEE International Conference on Computer Vision *Workshops* 2783–2788. doi: 10.1109/ICCVW.2017.327

[B86] Mellouk W. Handouzi W. (2020). Facial emotion recognition using deep learning: review and insights. Procedia Comput. Sci. 175, 689–694. doi: 10.1016/j.procs.2020.07.101

[B87] Mesquita B. (2001). Emotions in collectivist and individualist contexts. J. Pers. Soc. Psychol. 80:68. doi: 10.1037//0022-3514.80.1.68 11195892

[B88] Mesquita B. Frijda N. H. (1992). Cultural variations in emotions: a review. Psychol. Bull. 112:179. doi: 10.1037//0033-2909.112.2.179 1454891

[B89] Milone A. Cerniglia L. Cristofani C. Inguaggiato E. Levantini V. Masi G. . (2019). Empathy in youths with conduct disorder and callous-unemotional traits. Neural Plast. 2019:9638973. doi: 10.1155/2019/9638973 31097957 PMC6487083

[B90] Moran M. Gordon G. (2023). Deep curious feature selection: a recurrent, intrinsic-reward reinforcement learning approach to feature selection. IEEE Trans. Artif. Intell. 5, 1174–1184. doi: 10.1109/TAI.2023.3282564

[B91] Mourid M. R. Irfan H. Oduoye M. O. (2025). Artificial intelligence in pediatric epilepsy detection: balancing effectiveness with ethical considerations for welfare. Health Sci. Rep. 8:e70372. doi: 10.1002/hsr2.70372 39846037 PMC11751886

[B92] Nakisa B. (2019). Emotion Classification Using Advanced Machine Learning Techniques Applied to Wearable Physiological Signals Data. Brisbane: Queensland University of Technology.

[B93] Nakisa B. Rastgoo M. N. Tjondronegoro D. Chandran V. (2018). Evolutionary computation algorithms for feature selection of EEG-based emotion recognition using mobile sensors. Expert Syst. Appl. 93, 143–155. doi: 10.1016/j.eswa.2017.09.062

[B94] Nguyen D. Nguyen D. T. Sridharan S. Denman S. Nguyen T. T. Dean D. . (2023). Meta-transfer learning for emotion recognition. Neural Comput. Appl. 35, 10535–10549. doi: 10.1007/s00521-023-08248-y

[B95] Nguyen D. Nguyen K. Sridharan S. Abbasnejad I. Dean D. Fookes C. (2018). “Meta transfer learning for facial emotion recognition,” in 2018 24th International Conference on Pattern Recognition (ICPR) (Piscataway, NJ: IEEE), 3543–3548.

[B96] Nichol A. Achiam J. Schulman J. (2018). On first-order meta-learning algorithms. arXiv [Preprint]. arXiv:1803.02999. doi: 10.48550/arXiv.1803.02999

[B97] Nokelainen T. Airola P. Elnaggar M. S. I. (2023). Physiological signal-based emotion recognition from wearable devices (Master's thesis). University of Turku. Available online at: https://www.utupub.fi/handle/10024/174407 (Accessed February 17, 2023).

[B98] Oguz F. E. Alkan A. Schöler T. (2023). Emotion detection from ECG signals with different learning algorithms and automated feature engineering. Signal Image Video Process. 17, 3783–3791. doi: 10.1007/s11760-023-02606-y

[B99] Parisi G. I. Kemker R. Part J. L. Kanan C. Wermter S. (2019). Continual lifelong learning with neural networks: a review. Neural Netw. 113, 54–71. doi: 10.1016/j.neunet.2019.01.012 30780045

[B100] Parrott W. G. (2001). Emotions in Social Psychology: Essential Readings. Philadelphia, PA: Psychology Press.

[B101] Picard R. W. (2000). Affective Computing. Cambridge, MA: MIT Press.

[B102] Poszler F. Portmann E. Lütge C. (2025). Formalizing ethical principles within AI systems: experts' opinions on why (not) and how to do it. AI Ethics 5, 937–965. doi: 10.1007/s43681-024-00425-6

[B103] Pouyanfar S. Sadiq S. Yan Y. Tian H. Tao Y. Reyes M. P. . (2018). A survey on deep learning: algorithms, techniques, and applications. ACM Comput. Surv. 51, 1–36. doi: 10.1145/3234150

[B104] Pramod A. Naicker H. S. Tyagi A. K. (2021). “Machine learning and deep learning: open issues and future research directions for the next 10 years,” in Computational Analysis and Deep Learning for Medical Care: Principles, Methods, and Applications (Beverly, MA: Scrivener Publishing LLC), 463–490.

[B105] Qu F. Wang S.-J. Yan W.-J. Li H. Wu S. Fu X. (2017). CAS(ME)^2^: a database for spontaneous macro-expression and micro-expression spotting and recognition. IEEE Trans. Affect. Comput. 9, 424–436. doi: 10.1109/TAFFC.2017.2654440

[B106] Ramadan M. A. Salem N. M. Mahmoud L. N. Sadek I. (2024). Multimodal machine learning approach for emotion recognition using physiological signals. Biomed. Signal Process. Control 96:106553. doi: 10.1016/j.bspc.2024.106553

[B107] Ramírez-Arias F. J. García-Guerrero E. E. Tlelo-Cuautle E. Colores-Vargas J. M. García-Canseco E. López-Bonilla O. R. . (2022). Evaluation of machine learning algorithms for classification of EEG signals. Technologies 10:79. doi: 10.3390/technologies10040079

[B108] Rashmi C. Shantala C. (2022). EEG artifacts detection and removal techniques for brain computer interface applications: a systematic review. Int. J. Adv. Technol. Eng. Explor. 9:354. doi: 10.19101/IJATEE.2021.874883

[B109] Rastgoo M. N. Nakisa B. Maire F. Rakotonirainy A. Chandran V. (2019). Automatic driver stress level classification using multimodal deep learning. Expert Syst. Appl. 138:112793. doi: 10.1016/j.eswa.2019.07.010

[B110] Rastgoo M. N. Nakisa B. Rakotonirainy A. Maire F. Chandran V. (2024). Driver stress levels detection system using hyperparameter optimization. J. Intell. Transp. Syst. 28, 443–458. doi: 10.1080/15472450.2022.2140046

[B111] Raut N. (2018). Facial Emotion Recognition Using Machine Learning (Master's project). San Jose State University Scholar Works. Available online at: https://scholarworks.sjsu.edu/etd_projects/632 (Accessed Spring 2018).

[B112] Ravi R. Ramya V. J. Rani B. P. Nalluri S. Jenath M. (2025). “Ethical implications of emotion recognition technology in mental healthcare: navigating privacy, bias, and therapeutic boundaries,” in Explainable Artificial Intelligence in the Healthcare Industry (Beverly, MA: Wiley), 325–347.

[B113] Rothman N. B. Melwani S. (2017). Feeling mixed, ambivalent, and in flux: the social functions of emotional complexity for leaders. Acad. Manage. Rev. 42, 259–282. doi: 10.5465/amr.2014.0355

[B114] Russo D. P. Zorn K. M. Clark A. M. Zhu H. Ekins S. (2018). Comparing multiple machine learning algorithms and metrics for estrogen receptor binding prediction. Mol. Pharm. 15, 4361–4370. doi: 10.1021/acs.molpharmaceut.8b00546 30114914 PMC6181119

[B115] Saganowski S. Perz B. Polak A. G. Kazienko P. (2022). Emotion recognition for everyday life using physiological signals from wearables: a systematic literature review. IEEE Trans. Affect. Comput. 14, 1876–1897. doi: 10.1109/TAFFC.2022.3176135

[B116] Samadiani N. Huang G. Cai B. Luo W. Chi C.-H. Xiang Y. . (2019). A review on automatic facial expression recognition systems assisted by multimodal sensor data. Sensors 19:1863. doi: 10.3390/s19081863 31003522 PMC6514576

[B117] Sanchez E. Tellamekala M. K. Valstar M. Tzimiropoulos G. (2021). “Affective processes: stochastic modelling of temporal context for emotion and facial expression recognition,” in Proceedings of the IEEE/CVF Conference on Computer Vision and Pattern Recognition (Piscataway, NJ: IEEE), 9074–9084.

[B118] Sarkar P. Etemad A. (2020). Self-supervised ECG representation learning for emotion recognition. IEEE Trans. Affect. Comput. 13, 1541–1554. doi: 10.1109/TAFFC.2020.3014842

[B119] Scherer K. R. (2005). What are emotions? And how can they be measured? Soc. Sci. Inf. 44, 695–729. doi: 10.1177/0539018405058216

[B120] Schmidhuber J. (2015). Deep learning in neural networks: an overview. Neural Netw. 61, 85–117. doi: 10.1016/j.neunet.2014.09.003 25462637

[B121] Schulze P. Bestgen A.-K. Lech R. K. Kuchinke L. Suchan B. (2017). Preprocessing of emotional visual information in the human piriform cortex. Sci. Rep. 7:9191. doi: 10.1038/s41598-017-09295-x 28835658 PMC5569091

[B122] Sepúlveda A. Castillo F. Palma C. Rodriguez-Fernandez M. (2021). Emotion recognition from ECG signals using wavelet scattering and machine learning. Appl. Sci. 11:4945. doi: 10.3390/app11114945

[B123] Shoka A. Dessouky M. El-Sherbeny A. El-Sayed A. (2019). Literature review on EEG preprocessing, feature extraction, and classifications techniques. Menoufia J. Electron. Eng. Res. 28, 292–299. doi: 10.21608/mjeer.2019.64927

[B124] Shukla J. Barreda-Angeles M. Oliver J. Nandi G. C. Puig D. (2019). Feature extraction and selection for emotion recognition from electrodermal activity. IEEE Trans. Affect. Comput. 12, 857–869. doi: 10.1109/TAFFC.2019.2901673

[B125] Šimić G. Tkalčić M. Vukić V. Mulc D. Španić E. Šagud M. . (2021). Understanding emotions: origins and roles of the amygdala. Biomolecules 11:823. doi: 10.3390/biom11060823 34072960 PMC8228195

[B126] Soleymani M. Asghari-Esfeden S. Fu Y. Pantic M. (2015). Analysis of EEG signals and facial expressions for continuous emotion detection. IEEE Trans. Affect. Comput. 7, 17–28. doi: 10.1109/TAFFC.2015.2436926

[B127] Song H. Kim M. Park D. Shin Y. Lee J.-G. (2022). Learning from noisy labels with deep neural networks: a survey. IEEE Trans. Neural Netw. Learn. Syst. 34, 8135–8153. doi: 10.1109/TNNLS.2022.3152527 35254993

[B128] Sreeja P. S. Mahalakshmi G. (2017). Emotion models: a review. Int. J. Control Theory Appl. 10, 651–657.

[B129] Sun B. Lin Z. (2022). Emotion recognition using machine learning and ECG signals. arXiv [Preprint]. arXiv:2203.08477. doi: 10.48550/arXiv.2203.08477

[B130] Sun L. Song W. Han J. Yang L. (2025). Emotion recognition in consumers based on deep learning and image processing: applications in advertising. Trait. Signal 42:865. doi: 10.18280/ts.420223

[B131] Takalkar M. A. Xu M. (2017). “Image based facial micro-expression recognition using deep learning on small datasets,” in 2017 International Conference on Digital Image Computing: Techniques and Applications (DICTA) (Piscataway, NJ: IEEE), 1–7.

[B132] Tian S. Li L. Li W. Ran H. Ning X. Tiwari P. (2024). A survey on few-shot class-incremental learning. Neural Netw. 169, 307–324. doi: 10.1016/j.neunet.2023.10.039 37922714

[B133] Tripathi S. Acharya S. Sharma R. Mittal S. Bhattacharya S. (2017). “Using deep and convolutional neural networks for accurate emotion classification on DEAP data,” in Proceedings of the AAAI Conference on Artificial Intelligence (Palo Alto, CA: AAAI Press), 4746–4752.

[B134] Tzirakis P. Trigeorgis G. Nicolaou M. A. Schuller B. W. Zafeiriou S. (2017). End-to-end multimodal emotion recognition using deep neural networks. IEEE J. Sel. Top. Signal Process. 11, 1301–1309. doi: 10.1109/JSTSP.2017.2764438

[B135] Vempati R. Sharma L. D. (2023). A systematic review on automated human emotion recognition using electroencephalogram signals and artificial intelligence. Results Eng. 18:101027. doi: 10.1016/j.rineng.2023.101027

[B136] Wang F. He A. Liu C. Xiao W. Song Y. Chen C. . (2025). Physics-informed semi-supervised learning for hot-rolled strip flatness pattern recognition based on FixMatch method. Expert Syst. Appl. 296:128885. doi: 10.1016/j.eswa.2025.128885

[B137] Wang J. Tian Y. Yang Y. Chen X. Zheng C. Qiang W. (2024). Meta-auxiliary learning for micro-expression recognition. arXiv [Preprint]. arXiv:2404.12024. doi: 10.48550/arXiv.2404.12024

[B138] Wang K. Peng X. Yang J. Lu S. Qiao Y. (2020). “Suppressing uncertainties for large-scale facial expression recognition,” in Proceedings of the IEEE/CVF Conference on Computer Vision and Pattern *Recognition* 6897–6906. doi: 10.1109/CVPR42600.2020.00693

[B139] Wang W. Zhang J. Lin Z. Cui L. Zhang X. (2022). “Meta-learning improves emotion recognition,” in Proceedings of the World Conference on Intelligent and 3-D Technologies (WCI3DT 2022) Methods, Algorithms and Applications (Berlin: Springer), 13–22.

[B140] Wang X. Wang Y. Zhang D. (2023). Complex emotion recognition via facial expressions with label noises self-cure relation networks. Comput. Intell. Neurosci. 2023:7850140. doi: 10.1155/2023/7850140 36711195 PMC9879680

[B141] Wang X.-W. Nie D. Lu B.-L. (2014). Emotional state classification from EEG data using machine learning approach. Neurocomputing 129, 94–106. doi: 10.1016/j.neucom.2013.06.046

[B142] Wang Y. Gao Z. Zhang J. Cao X. Zheng D. Gao Y. . (2021). Trajectory design for UAV-based Internet of Things data collection: a deep reinforcement learning approach. IEEE Internet Things J. 9, 3899–3912. doi: 10.1109/JIOT.2021.3102185

[B143] Wang Y. Song W. Tao W. Liotta A. Yang D. Li X. . (2022). A systematic review on affective computing: emotion models, databases, and recent advances. Inf. Fusion 83, 19–52. doi: 10.1016/j.inffus.2022.03.009

[B144] Wang Z. Hu G. Hu Q. (2020). “Training noise-robust deep neural networks via meta-learning,” in Proceedings of the IEEE/CVF Conference on Computer Vision and Pattern Recognition (Piscataway, NJ: IEEE), 4524–4533.

[B145] Wang Z. Wang Y. (2025). Emotion recognition based on multimodal physiological electrical signals. Front. Neurosci. 19:1512799. doi: 10.3389/fnins.2025.1512799 40109659 PMC11919864

[B146] Weatherall A. Robles J. S. (2021). “How emotions are made to do things: an introduction,” in How Emotions are Made in Talk (Amsterdam: John Benjamins Publishing Company), 1–24.

[B147] Wierzbicka A. (1999). Emotions Across Languages and Cultures: Diversity and Universals. Cambridge: Cambridge University Press.

[B148] Wu X. He R. Sun Z. Tan T. (2018). A light CNN for deep face representation with noisy labels. IEEE Trans. Inf. Forensics Secur. 13, 2884–2896. doi: 10.1109/TIFS.2018.2833032

[B149] Wu Y. Mi Q. Gao T. (2025). A comprehensive review of multimodal emotion recognition: techniques, challenges, and future directions. Biomimetics 10:418. doi: 10.3390/biomimetics10070418 40710231 PMC12292624

[B150] Wu Y.-C. Chiu L.-W. Lai C.-C. Wu B.-F. Lin S. S. (2023). Recognizing, fast and slow: complex emotion recognition with facial expression detection and remote physiological measurement. IEEE Trans. Affect. Comput. 14, 3177–3190. doi: 10.1109/TAFFC.2023.3253859

[B151] Xia Z. Hong X. Gao X. Feng X. Zhao G. (2019). Spatiotemporal recurrent convolutional networks for recognizing spontaneous micro-expressions. IEEE Trans. Multimedia 22, 626–640. doi: 10.1109/TMM.2019.2931351

[B152] Xia Z. Huang H. Chen H. Feng X. Zhao G. (2025). Hybrid-supervised hypergraph-enhanced transformer for micro-gesture based emotion recognition. IEEE Trans. Affect. Comput. doi: 10.1109/TAFFC.2025.3618639. [Epub ahead of print].

[B153] Xia Z. Peng W. Khor H.-Q. Feng X. Zhao G. (2020). Revealing the invisible with model and data shrinking for composite-database micro-expression recognition. IEEE Trans. Image Process. 29, 8590–8605. doi: 10.1109/TIP.2020.3018222 32845838

[B154] Yan H. Gu Y. Zhang X. Wang Y. Ji Y. Ren F. (2022). “Mitigating label-noise for facial expression recognition in the wild,” in 2022 IEEE International Conference on Multimedia and Expo (ICME) (Piscataway, NJ: IEEE), 1–6.

[B155] Yan W.-J. Li X. Wang S.-J. Zhao G. Liu Y.-J. Chen Y.-H. . (2014). CASME II: an improved spontaneous micro-expression database and the baseline evaluation. PLoS ONE 9:e86041. doi: 10.1371/journal.pone.0086041 24475068 PMC3903513

[B156] Yan W.-J. Wu Q. Liu Y.-J. Wang S.-J. Fu X. (2013). “CASME database: a dataset of spontaneous micro-expressions collected from neutralized faces,” in 2013 10th IEEE International Conference and Workshops on Automatic Face and Gesture Recognition (FG) (Piscataway, NJ: IEEE), 1–7.

[B157] Yang K. Wang C. Gu Y. Sarsenbayeva Z. Tag B. Dingler T. . (2021). Behavioral and physiological signals-based deep multimodal approach for mobile emotion recognition. IEEE Trans. Affect. Comput. 14, 1082–1097. doi: 10.1109/TAFFC.2021.3100868

[B158] Zahara L. Musa P. Wibowo E. P. Karim I. Musa S. B. (2020). “The facial emotion recognition (FER-2013) dataset for prediction system of micro-expressions face using the convolutional neural network (CNN) algorithm based raspberry pi,” in 2020 Fifth International Conference on Informatics and Computing (ICIC) (Piscataway, NJ: IEEE), 1–9.

[B159] Zeng J. Shan S. Chen X. (2018). “Facial expression recognition with inconsistently annotated datasets,” in Proceedings of the European Conference on Computer Vision (Cham: Springer), 222–237.

[B160] Zhang F. Liu Y. Yu X. Wang Z. Zhang Q. Wang J. . (2025). Towards facial micro-expression detection and classification using modified multimodal ensemble learning approach. Inf. Fusion 115:102735. doi: 10.1016/j.inffus.2024.102735

[B161] Zhang K. Li Y. Wang J. Cambria E. Li X. (2021). Real-time video emotion recognition based on reinforcement learning and domain knowledge. IEEE Trans. Circuits Syst. Video Technol. 32, 1034–1047. doi: 10.1109/TCSVT.2021.3072412

[B162] Zhang Y. Chen J. Tan J. H. Chen Y. Chen Y. Li D. . (2020). An investigation of deep learning models for EEG-based emotion recognition. Front. Neurosci. 14:622759. doi: 10.3389/fnins.2020.622759 33424547 PMC7785875

[B163] Zhao J. Mao X. Chen L. (2019). Speech emotion recognition using deep 1D & 2D CNN LSTM networks. Biomed. Signal Process. Control 47, 312–323. doi: 10.1016/j.bspc.2018.08.035

[B164] Zhao K. Liu X. Yang G. (2025). M3ENet: a multi-modal fusion network for efficient micro-expression recognition. Sensors 25:6276. doi: 10.3390/s25206276 41157326 PMC12568106

[B165] Zhao Y. Xu J. (2019). A convolutional neural network for compound micro-expression recognition. Sensors 19:5553. doi: 10.3390/s19245553 31888182 PMC6960609

[B166] Zheng Q. Tian X. Yu Z. Yang M. Elhanashi A. Saponara S. (2025). Robust automatic modulation classification using asymmetric trilinear attention net with noisy activation function. Eng. Appl. Artif. Intell. 141:109861. doi: 10.1016/j.engappai.2024.109861

[B167] Zhou F. Cao C. Zhong T. Geng J. (2021). Learning meta-knowledge for few-shot image emotion recognition. Expert Syst. Appl. 168:114274. doi: 10.1016/j.eswa.2020.114274

[B168] Zhu C. Li P. Zhang Z. Liu D. Luo W. (2019). Characteristics of the regulation of the surprise emotion. Sci. Rep. 9:7576. doi: 10.1038/s41598-019-42951-y 31110212 PMC6527688

[B169] Zhu X. Liu Z. Cambria E. Yu X. Fan X. Chen H. . (2025). A client–server based recognition system: non-contact single/multiple emotional and behavioral state assessment methods. Comput. Methods Programs Biomed. 260:108564. doi: 10.1016/j.cmpb.2024.108564 39732086

