# Supplementary material for: Complex emotion recognition system using basic emotions via facial expression, electroencephalogram, and electrocardiogram signals: a review
Source: Front Psychol. 2026 Mar 2;17:1682883. doi: 10.3389/fpsyg.2026.1682883 (PMC12989340; doi:10.3389/fpsyg.2026.1682883)
Supplement: Supplementary file 1 [file Table_1.docx]

**Supplementary Material A: Search Strategy and Study Selection (PRISMA)**

**A.1 Databases and Time Span**

A systematic literature search was conducted across the following electronic databases: IEEE Xplore, ScienceDirect, SpringerLink, Wiley Online Library, and Google Scholar. The search covered studies published between January 2017 and December 2024.

**A.2 Search Terms and Strategy**

Search queries were designed to capture studies related to complex emotion recognition systems (CERS) and meta-learning–based approaches. Database-specific adaptations were applied where necessary. The core search string was:

(“complex emotion recognition” OR “emotion recognition” OR “affective computing” OR “multimodal emotion”) AND (“meta-learning” OR “few-shot learning” OR “continual learning” OR “reinforcement learning” OR “label noise”)

Examples of database-specific implementations include:

- **ScienceDirect / Springer / Wiley:**Title, abstract, and keyword search using the core string.
- **Google Scholar:**

Broad keyword search with relevance-based screening of the first several hundred results.

The complete search strings for each database are provided in this Supplement.

**A.3 Inclusion and Exclusion Criteria**

**Inclusion criteria:**

- Peer-reviewed journal articles or conference papers
- Studies addressing complex or multimodal emotion recognition
- Use of AI- or machine-learning–based approaches, including meta-learning paradigms
- Publications in English

**Exclusion criteria:**

- Non-peer-reviewed articles, editorials, or abstracts only
- Studies focused solely on basic emotion recognition without complexity or multimodality
- Papers lacking methodological details or experimental validation

**A.4 Screening and Selection Process**

After removal of duplicate and irrelevant records, titles and abstracts were screened for relevance. Full-text assessment was subsequently conducted to evaluate eligibility based on the predefined criteria. Disagreements during screening were resolved through discussion among the authors.

**A.5 Quality Assessment**

Given the heterogeneity of datasets, modalities, and evaluation protocols, a formal quantitative quality scoring was not applied. Instead, methodological soundness, dataset description, and clarity of evaluation metrics were qualitatively assessed to ensure relevance and rigor.

**A.6 PRISMA Flow Diagram**

The study selection process, including identification, screening, eligibility, and inclusion stages, is summarized in Figure 1 following the PRISMA guidelines.


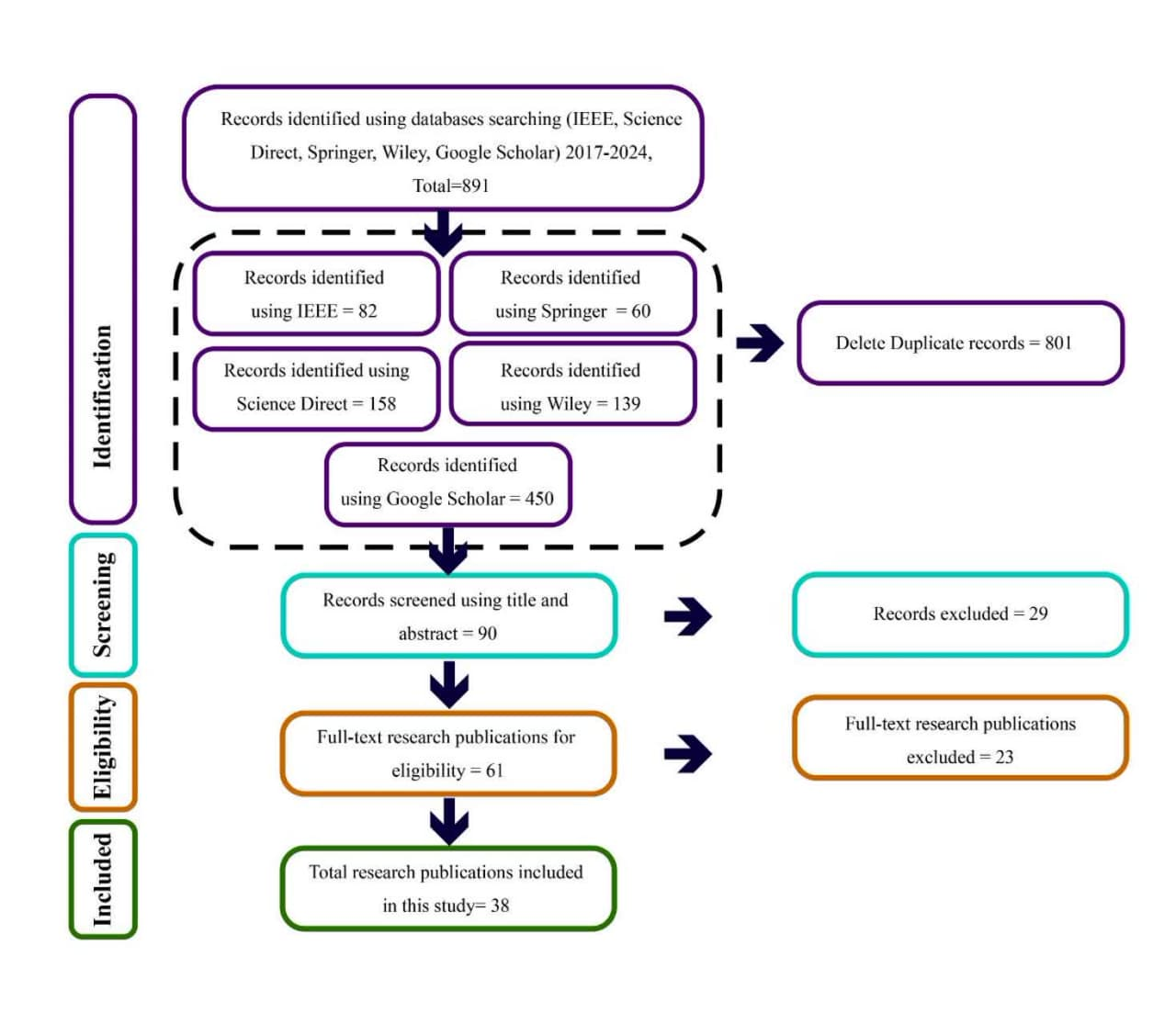


**Figure 1.** PRISMA flow diagram illustrating the identification, screening, eligibility assessment, and inclusion of studies in this review.
